# Supplementary figures and images for: Collaborative Cross mice have diverse phenotypic responses to infection with Methicillin-resistant Staphylococcus aureus USA300
Source: PLoS Genet. 2024 May 2;20(5):e1011229. doi: 10.1371/journal.pgen.1011229 (PMC11108197; doi:10.1371/journal.pgen.1011229)

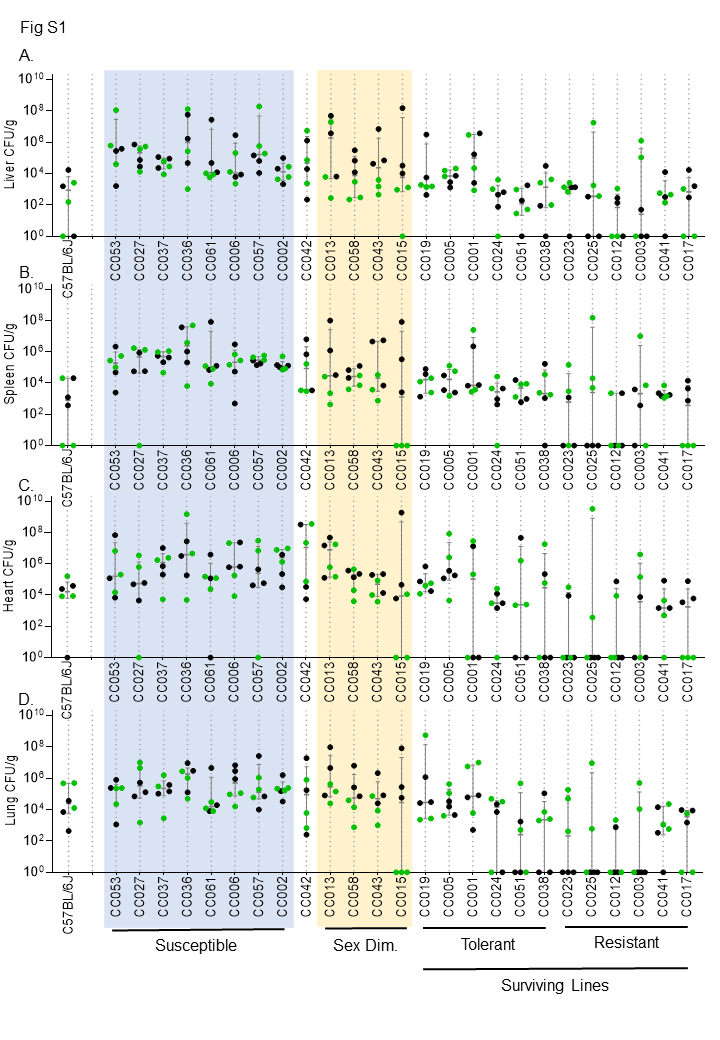

Supplement: S1 Fig — A. Liver colonization. B. spleen colonization, C. heart colonization. D. lung colonization. Strains are shown in ascending order of survival. Where survival was equal, strains are arranged in descending order of total organ colonization. Dots represent individual mice; black dots represent males; green dots represent females. The median and interquartile range are shown for each strain. (TIF) [file pgen.1011229.s001.TIF]

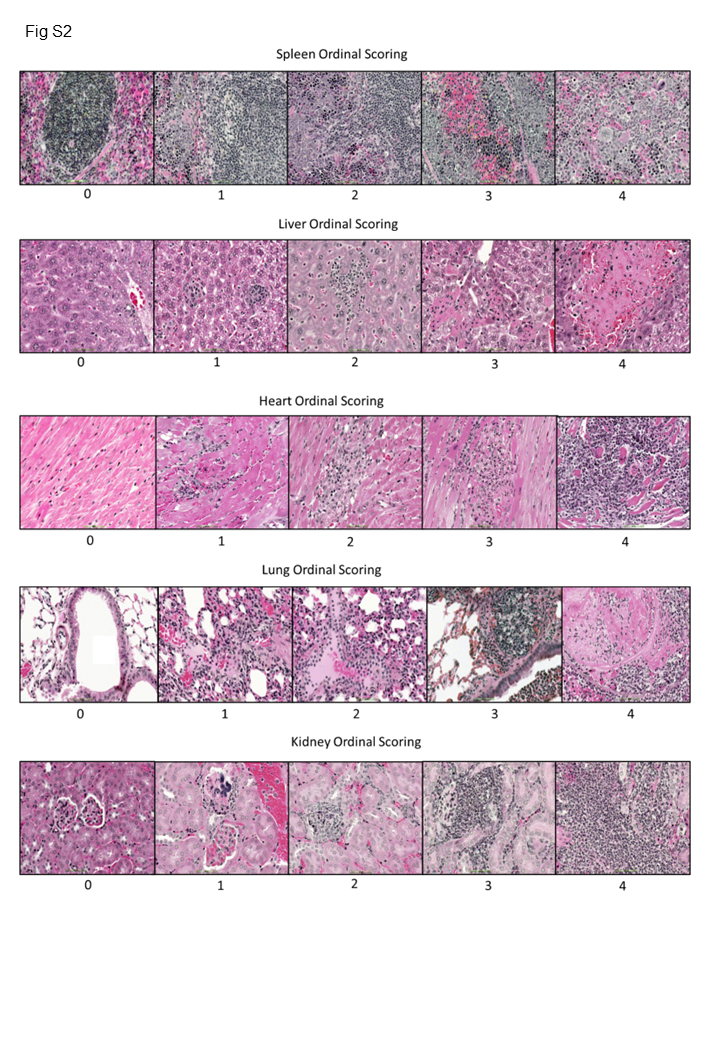

Supplement: S2 Fig — Tissues were sectioned and stained with H&E for scoring. Shows the 400X magnification of representative scoring for all the organs. (TIF) [file pgen.1011229.s002.TIF]

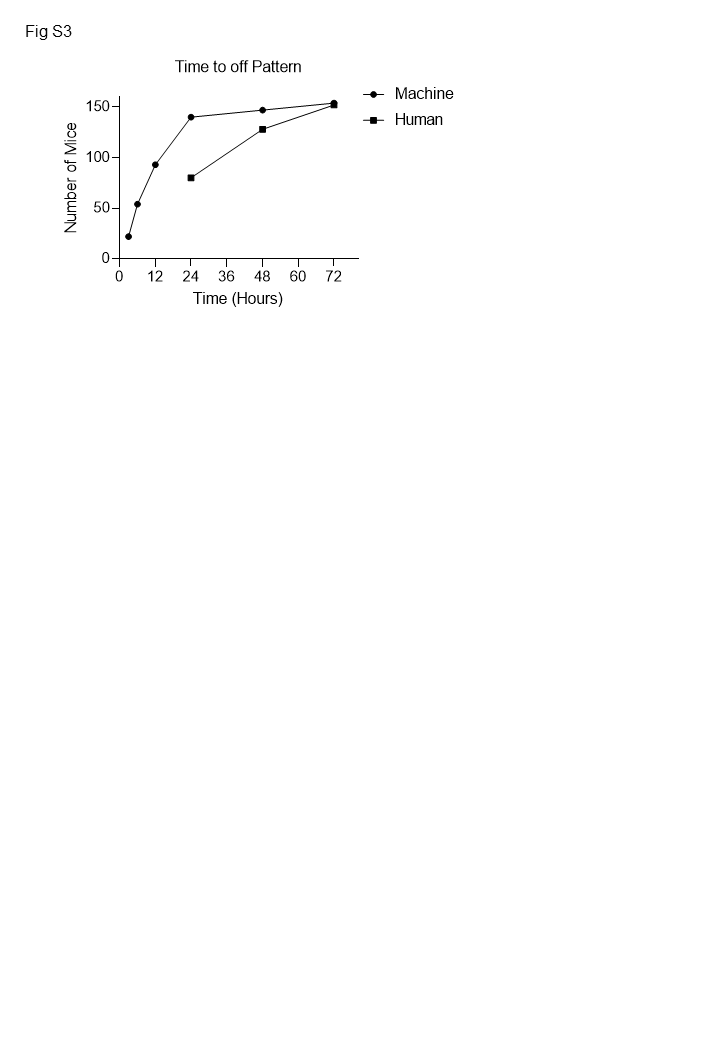

Supplement: S3 Fig — Plot showing the number of animals that deviated from their normal pattern as identified by telemetry system (machine—dot) vs. Laboratory worker (human—squares). (TIF) [file pgen.1011229.s003.TIF]

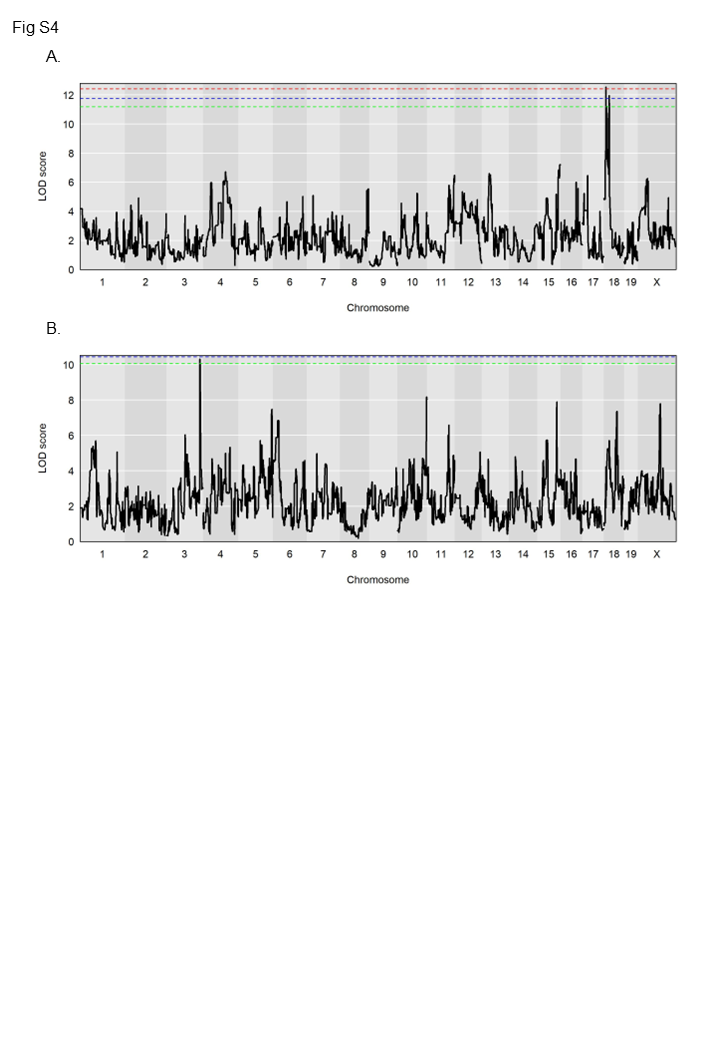

Supplement: S4 Fig — LOD plots for square root transformed survival phenotype excluding the four sexually dimorphic strains. A. Early susceptibility. B. Late survival. The dotted (Red– 99%, Blue– 95%, Green– 90%) lines represent the significant LOD scores for 999 permutations. (TIF) [file pgen.1011229.s004.TIF]

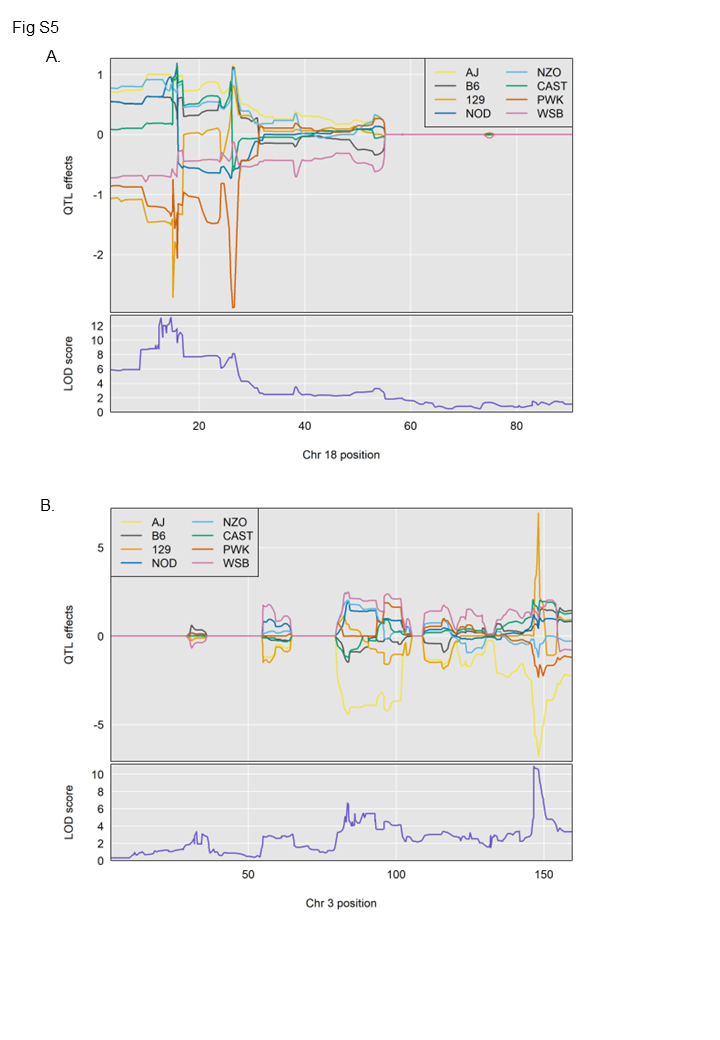

Supplement: S5 Fig — A. ESMI peak (day 2 post-infection) on chromosome 18. B. LSMI peak (day 7 post-infection) on chromosome 3. (TIF) [file pgen.1011229.s005.TIF]

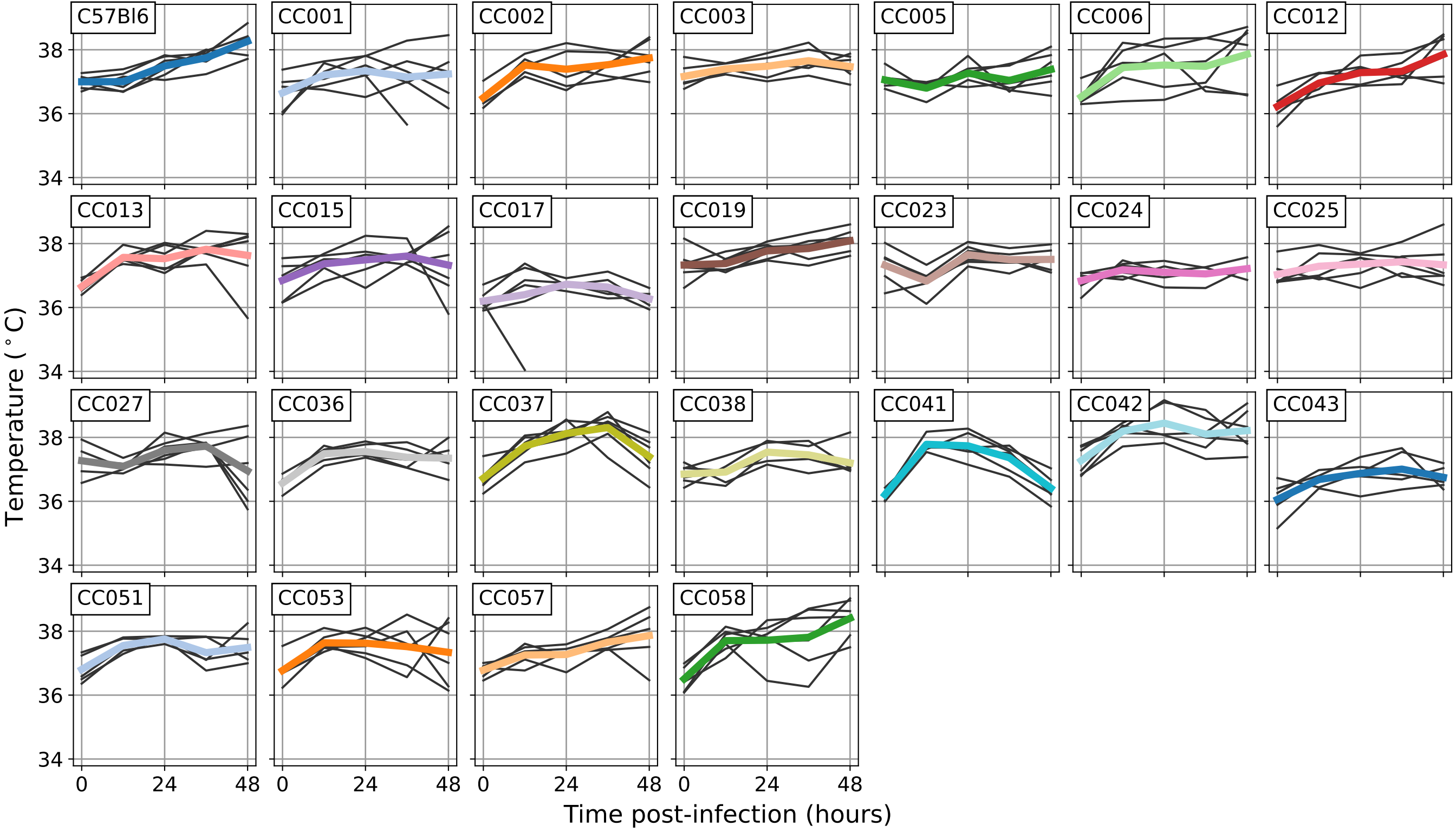

Supplement: S7 Fig — Change in temperature for each mouse after infection (for 48 hours). The data is grouped by CC strain and colored lines in each plot represent the strain averages. (PDF) [file pgen.1011229.s007.pdf]
