# Supplementary material for: Collaborative Cross mice have diverse phenotypic responses to infection with Methicillin-resistant Staphylococcus aureus USA300
Source: PLoS Genet. 2024 May 2;20(5):e1011229. doi: 10.1371/journal.pgen.1011229 (PMC11108197; doi:10.1371/journal.pgen.1011229)
Supplement: S6 Fig — Blue line represents temperature, black line represents activity and red line represents the time of infection. (PDF) [file pgen.1011229.s006.pdf]

Supplementary Fig S8

Circadian pattern for all mice involved in the study. Blue line represents temperature, black line represents activity and red line represents the time of infection.

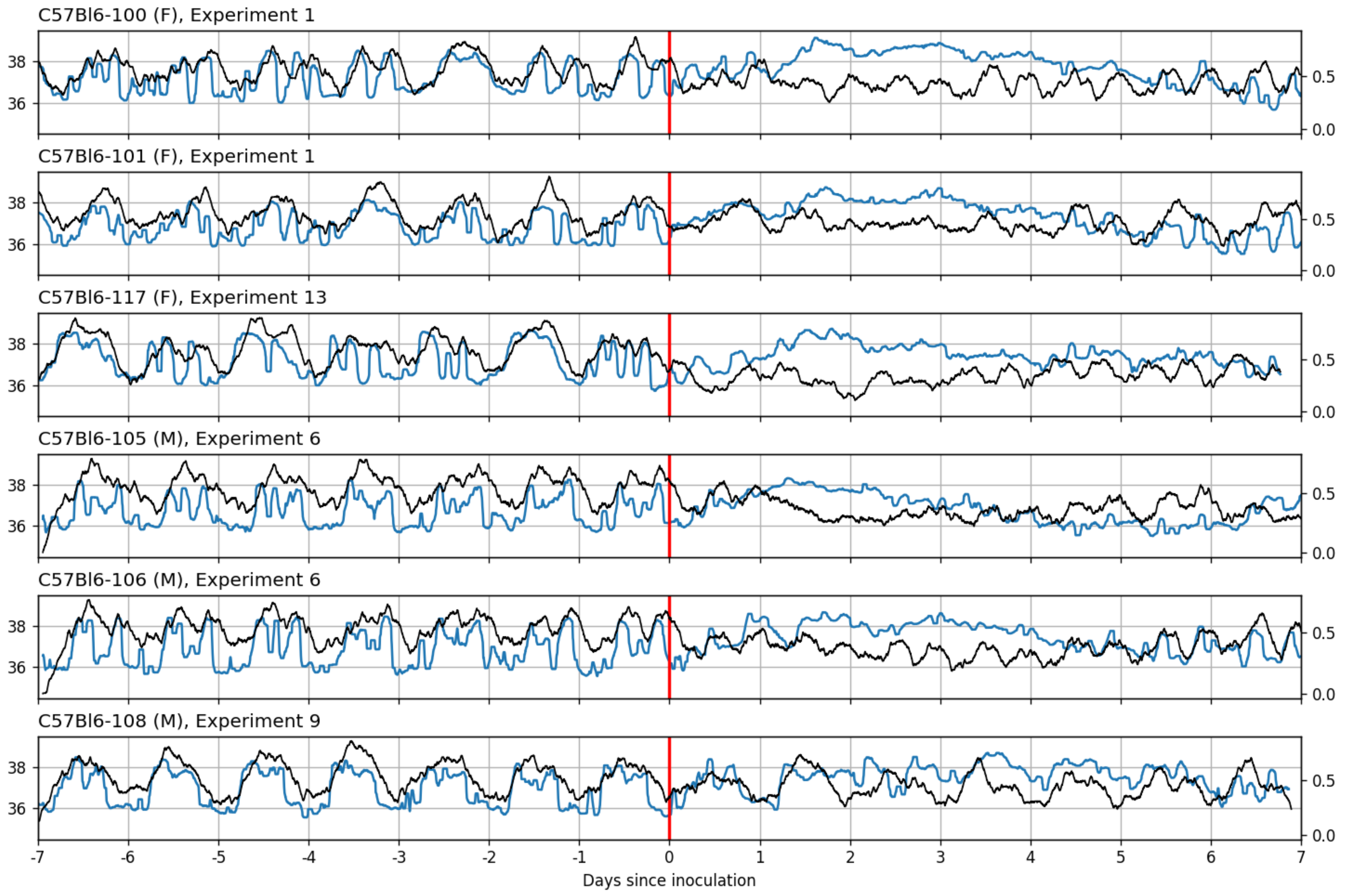

C57Bl6-100 (F), Experiment 1
C57Bl6-101 (F), Experiment 1
C57Bl6-117 (F), Experiment 13
C57Bl6-105 (M), Experiment 6
C57Bl6-106 (M), Experiment 6
C57Bl6-108 (M), Experiment 9
38
36
0.5
0.0
-7
-6
-5
-4
-3
-2
-1
0
1
2
3
4
5
6
7
Days since inoculation

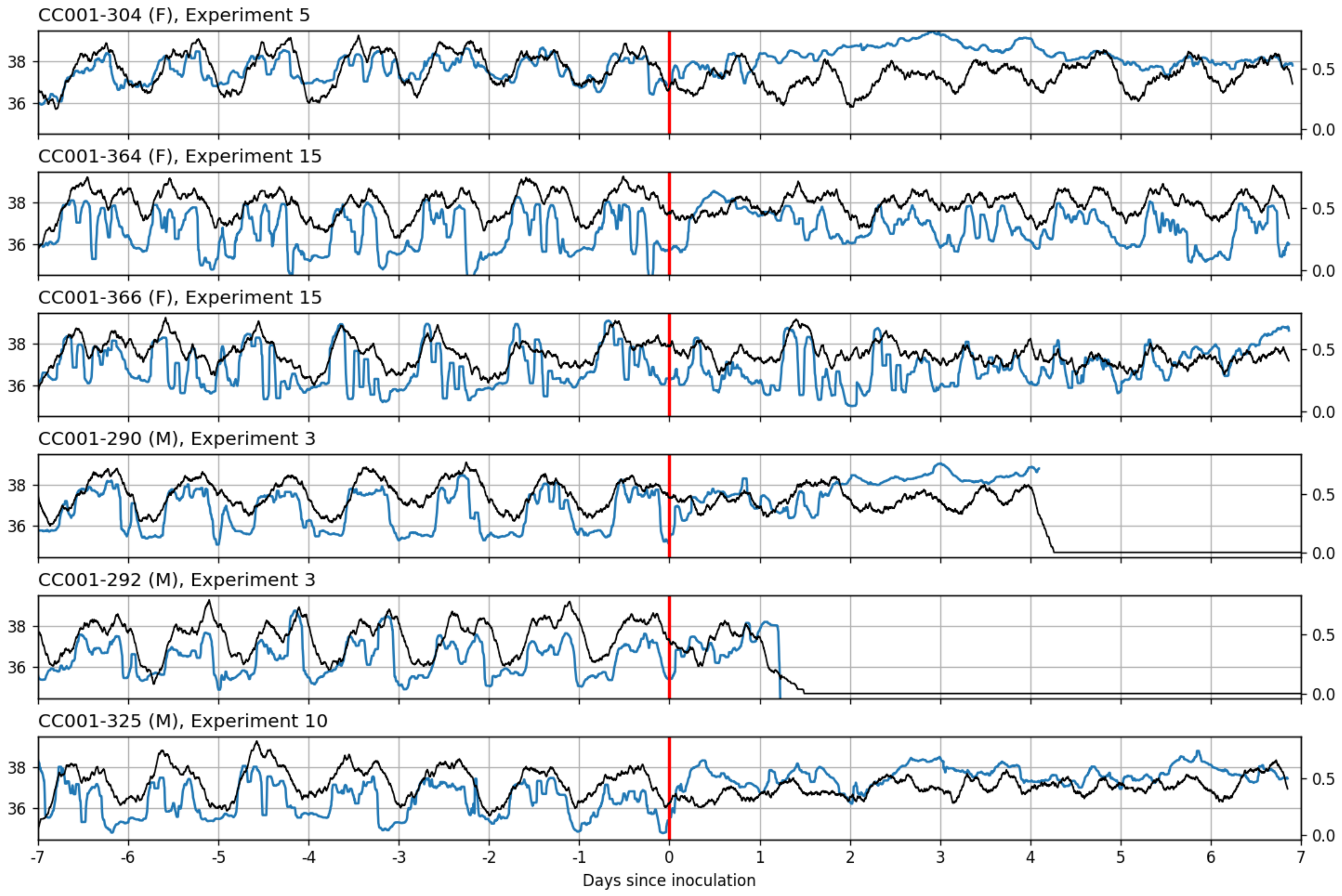

CC001-304 (F), Experiment 5
CC001-364 (F), Experiment 15
CC001-366 (F), Experiment 15
CC001-290 (M), Experiment 3
CC001-292 (M), Experiment 3
CC001-325 (M), Experiment 10
38
36
0.5
0.0
-7
-6
-5
-4
-3
-2
-1
0
1
2
3
4
5
6
7
Days since inoculation

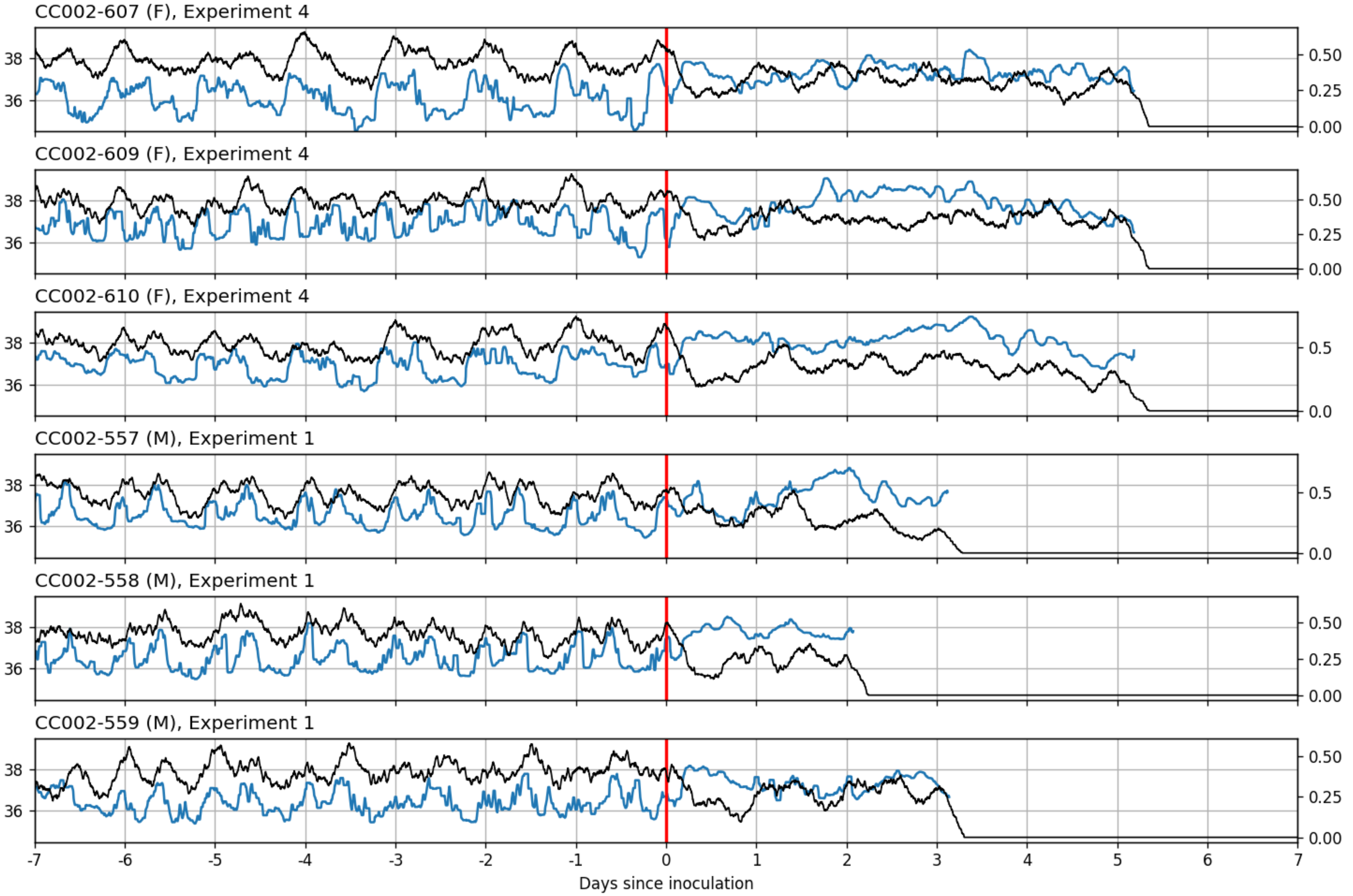

CC002-607 (F), Experiment 4
CC002-609 (F), Experiment 4
CC002-610 (F), Experiment 4
CC002-557 (M), Experiment 1
CC002-558 (M), Experiment 1
CC002-559 (M), Experiment 1
36
38
0.00
0.25
0.50
0.0
0.5
-7
-6
-5
-4
-3
-2
-1
0
1
2
3
4
5
6
7
Days since inoculation

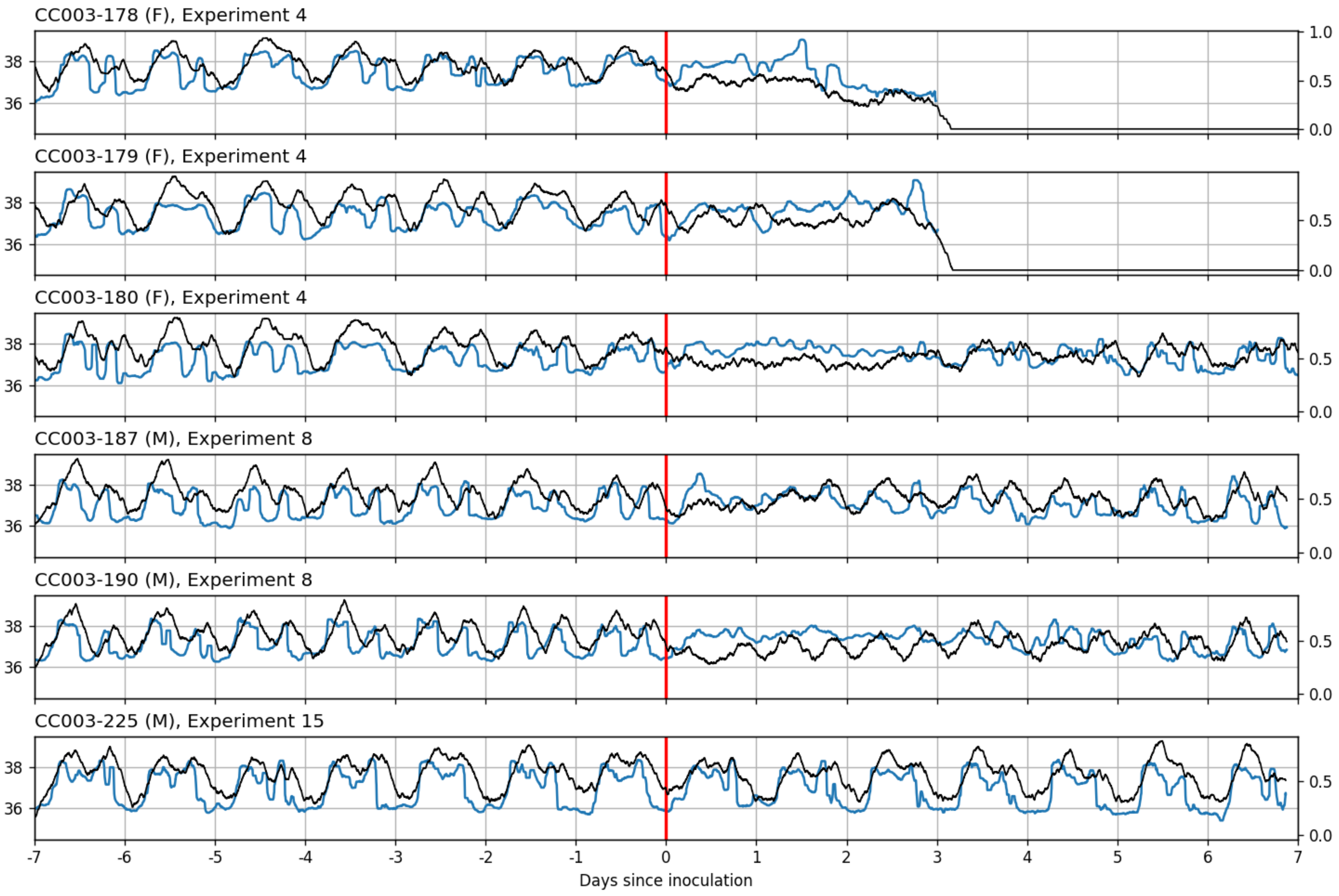

CC003-178 (F), Experiment 4
CC003-179 (F), Experiment 4
CC003-180 (F), Experiment 4
CC003-187 (M), Experiment 8
CC003-190 (M), Experiment 8
CC003-225 (M), Experiment 15
36
38
0.0
0.5
1.0
-7
-6
-5
-4
-3
-2
-1
0
1
2
3
4
5
6
7
Days since inoculation

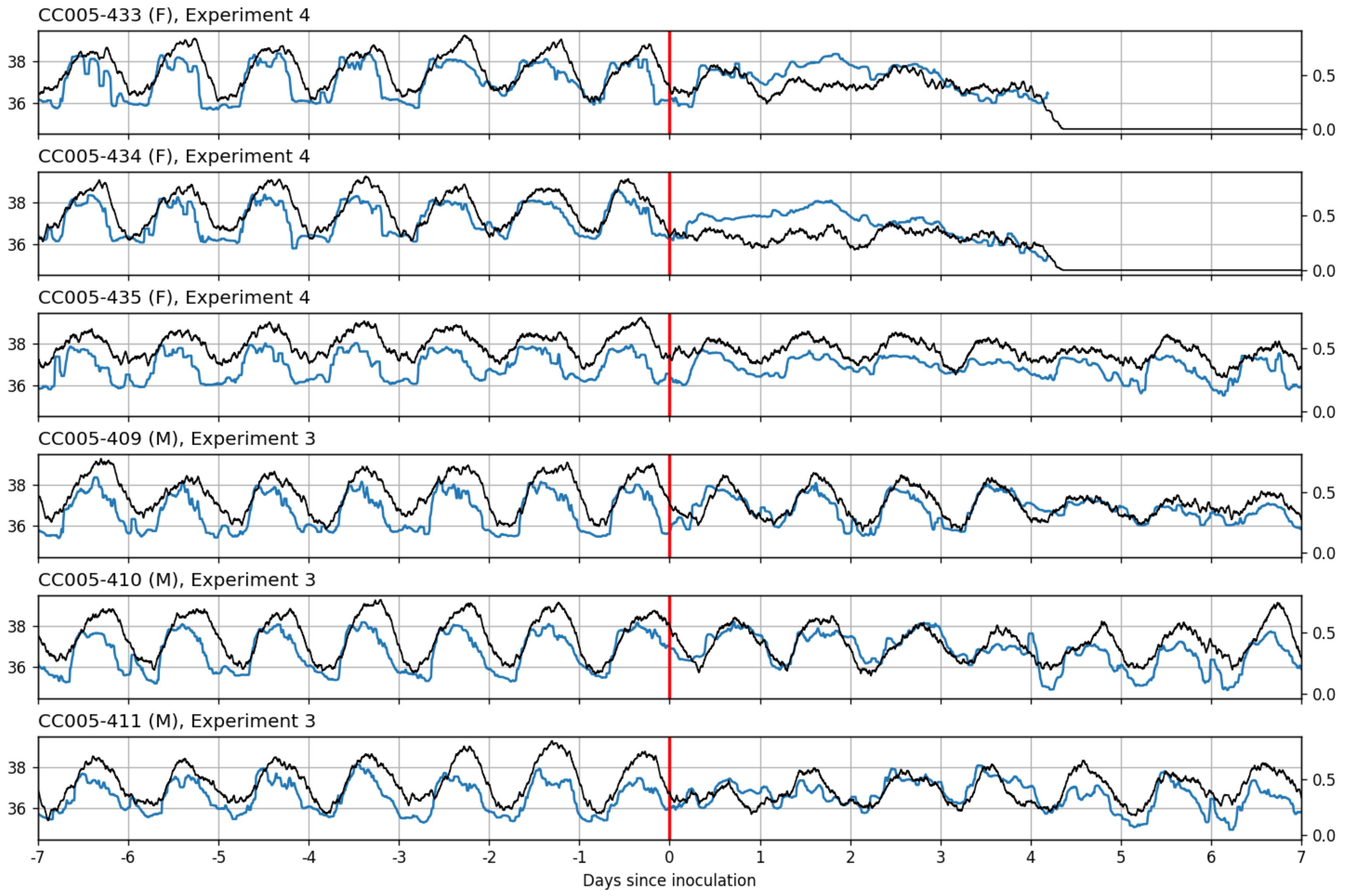
CC005-433 (F), Experiment 4
CC005-434 (F), Experiment 4
CC005-435 (F), Experiment 4
CC005-409 (M), Experiment 3
CC005-410 (M), Experiment 3
CC005-411 (M), Experiment 3
38
36
0.5
0.0
-7
-6
-5
-4
-3
-2
-1
0
1
2
3
4
5
6
7
Days since inoculation

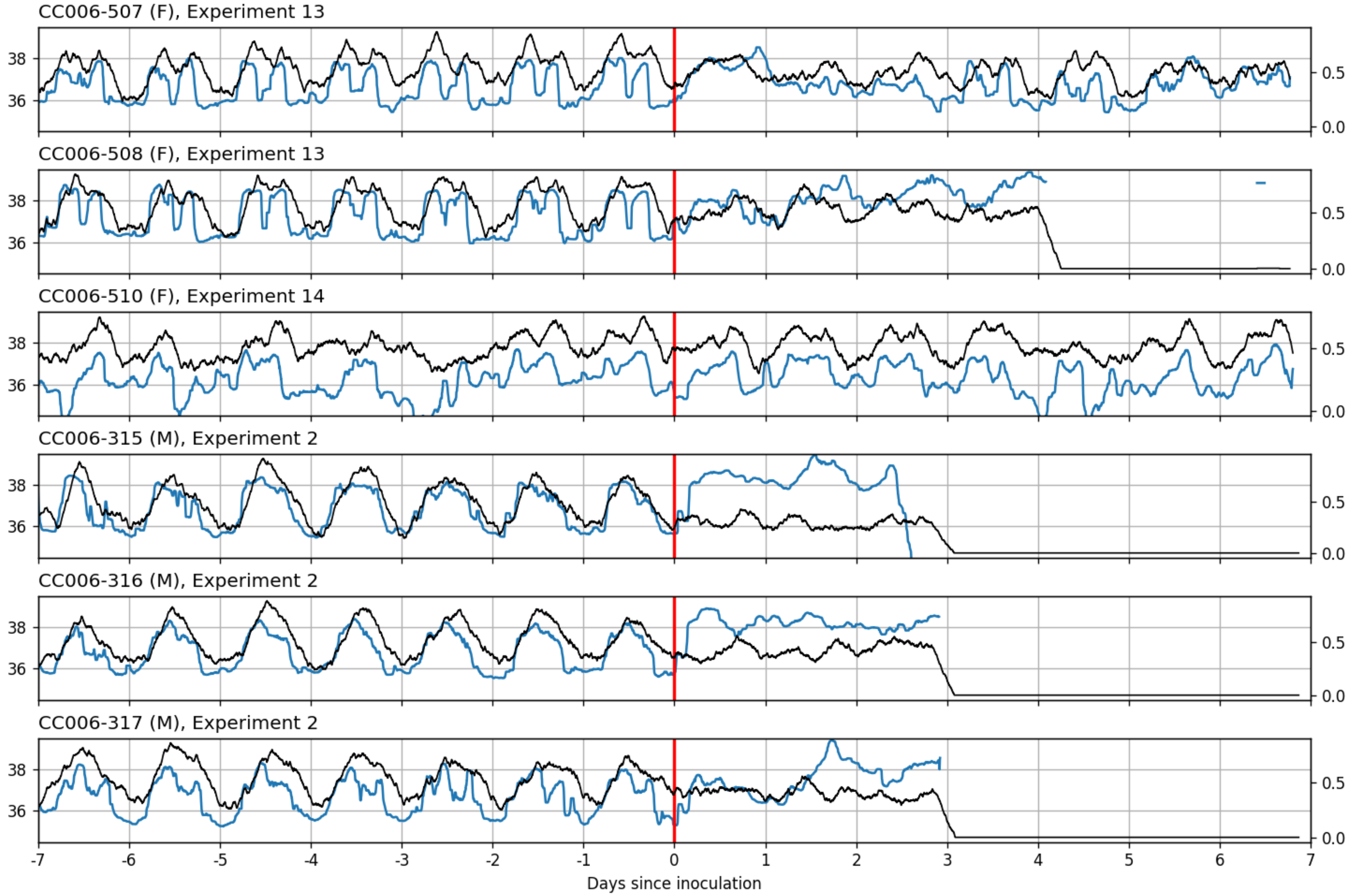

CC006-507 (F), Experiment 13
CC006-508 (F), Experiment 13
CC006-510 (F), Experiment 14
CC006-315 (M), Experiment 2
CC006-316 (M), Experiment 2
CC006-317 (M), Experiment 2
38
36
0.5
0.0
-7
-6
-5
-4
-3
-2
-1
0
1
2
3
4
5
6
7
Days since inoculation

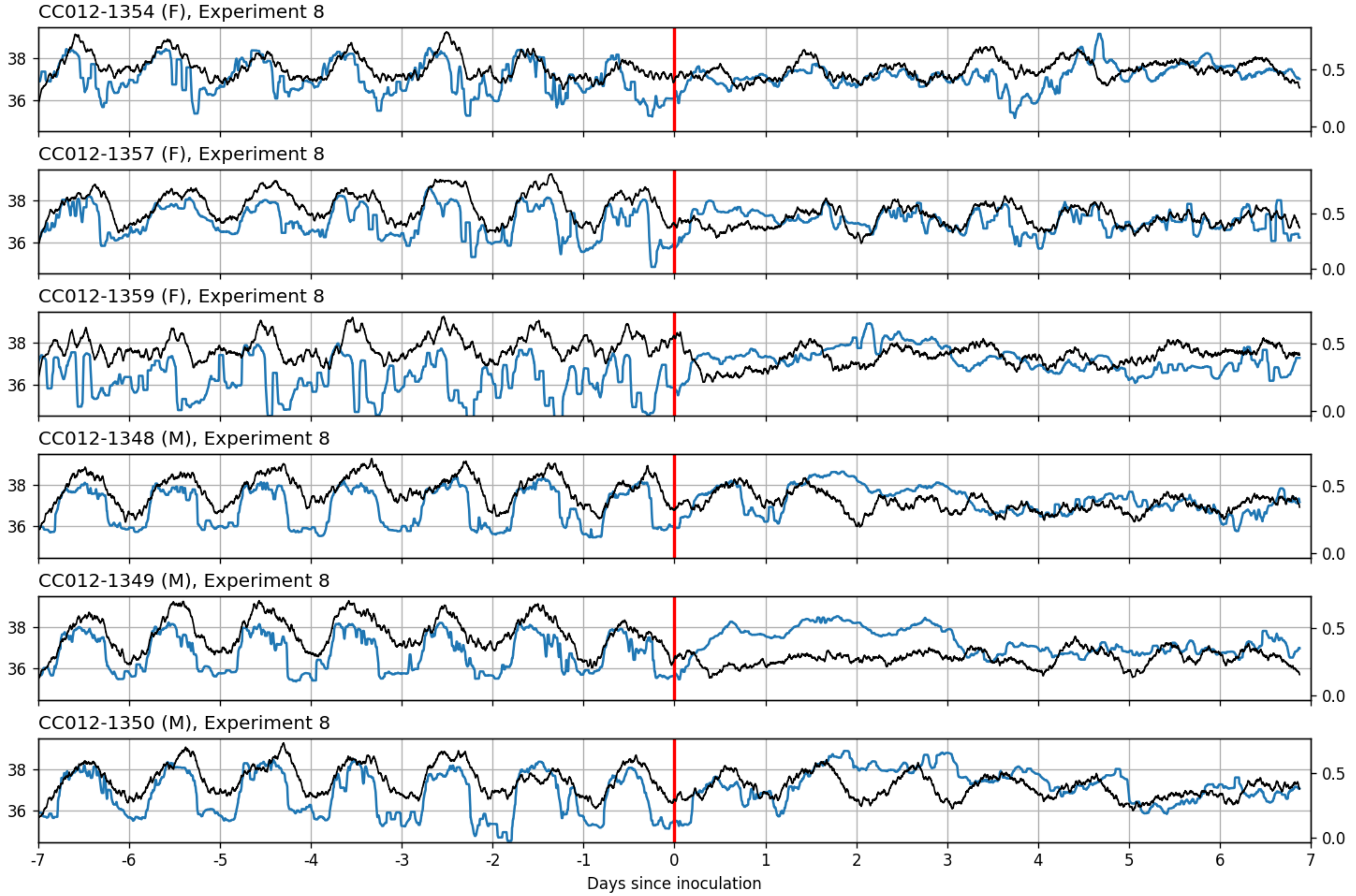

CC012-1354 (F), Experiment 8
CC012-1357 (F), Experiment 8
CC012-1359 (F), Experiment 8
CC012-1348 (M), Experiment 8
CC012-1349 (M), Experiment 8
CC012-1350 (M), Experiment 8
38
36
0.5
0.0
-7
-6
-5
-4
-3
-2
-1
0
1
2
3
4
5
6
7
Days since inoculation

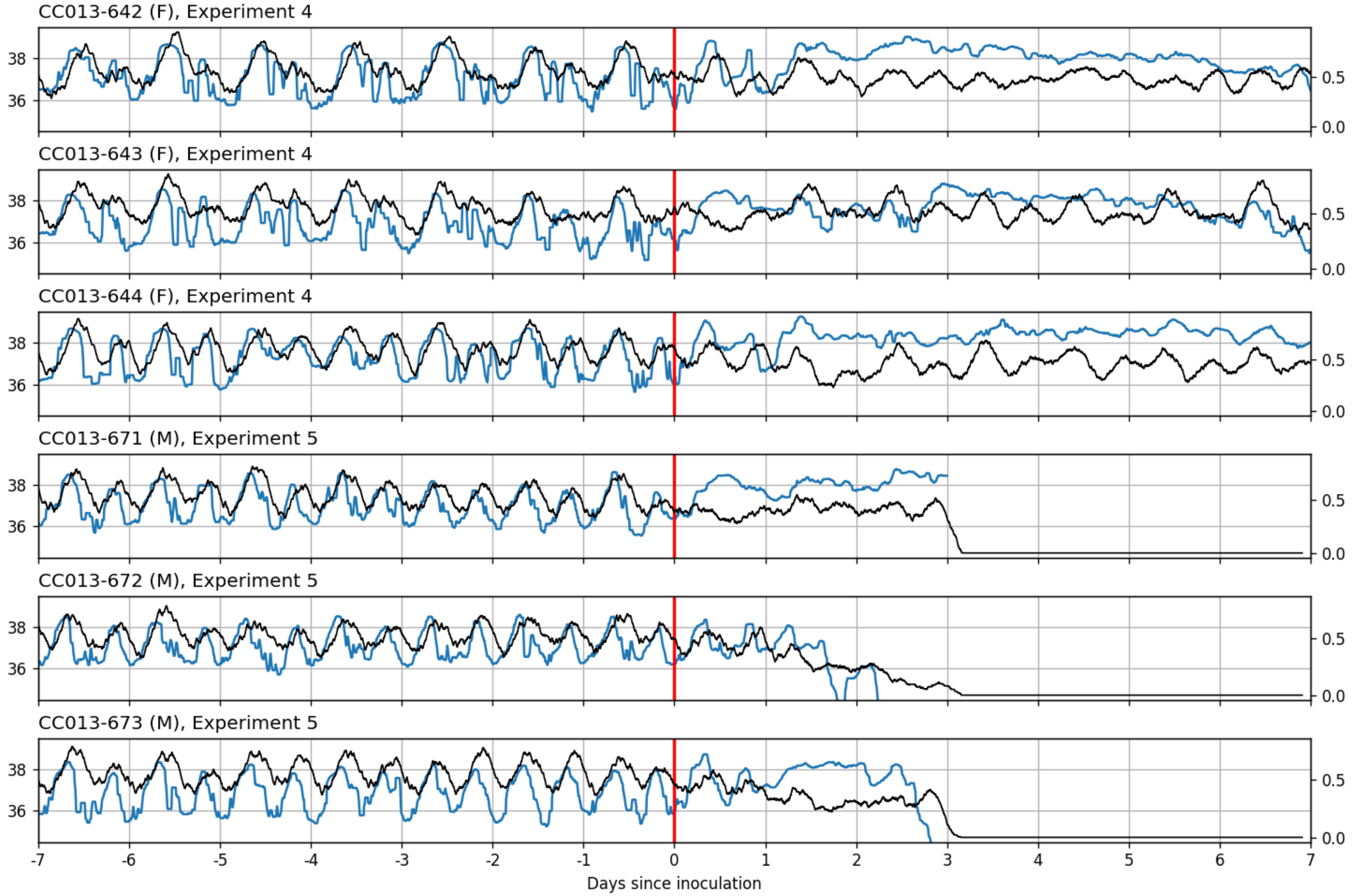

CC013-642 (F), Experiment 4
CC013-643 (F), Experiment 4
CC013-644 (F), Experiment 4
CC013-671 (M), Experiment 5
CC013-672 (M), Experiment 5
CC013-673 (M), Experiment 5
38
36
0.5
0.0
-7
-6
-5
-4
-3
-2
-1
0
1
2
3
4
5
6
7
Days since inoculation

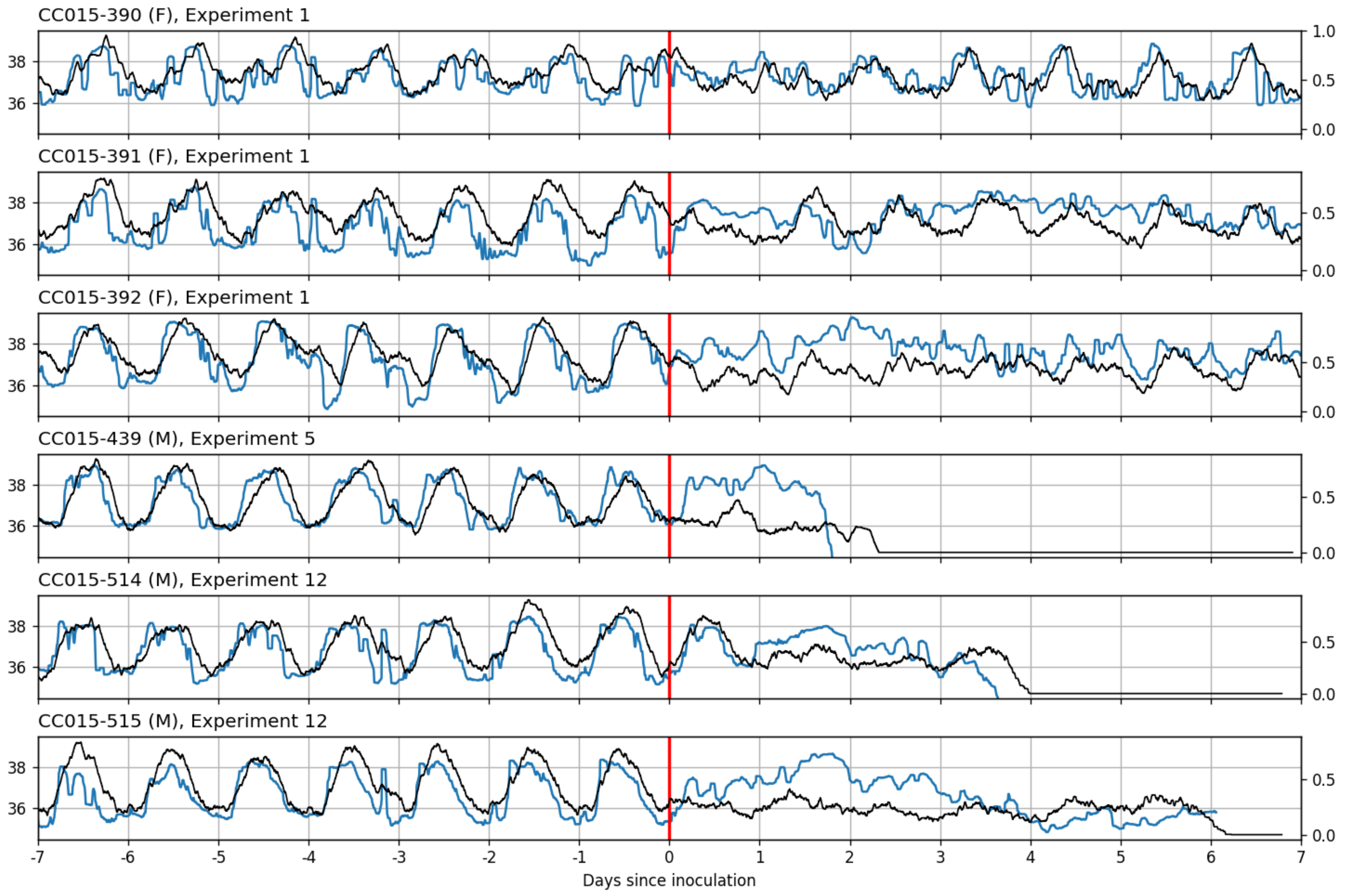

CC015-390 (F), Experiment 1
CC015-391 (F), Experiment 1
CC015-392 (F), Experiment 1
CC015-439 (M), Experiment 5
CC015-514 (M), Experiment 12
CC015-515 (M), Experiment 12
36
38
0.0
0.5
1.0
-7
-6
-5
-4
-3
-2
-1
0
1
2
3
4
5
6
7
Days since inoculation

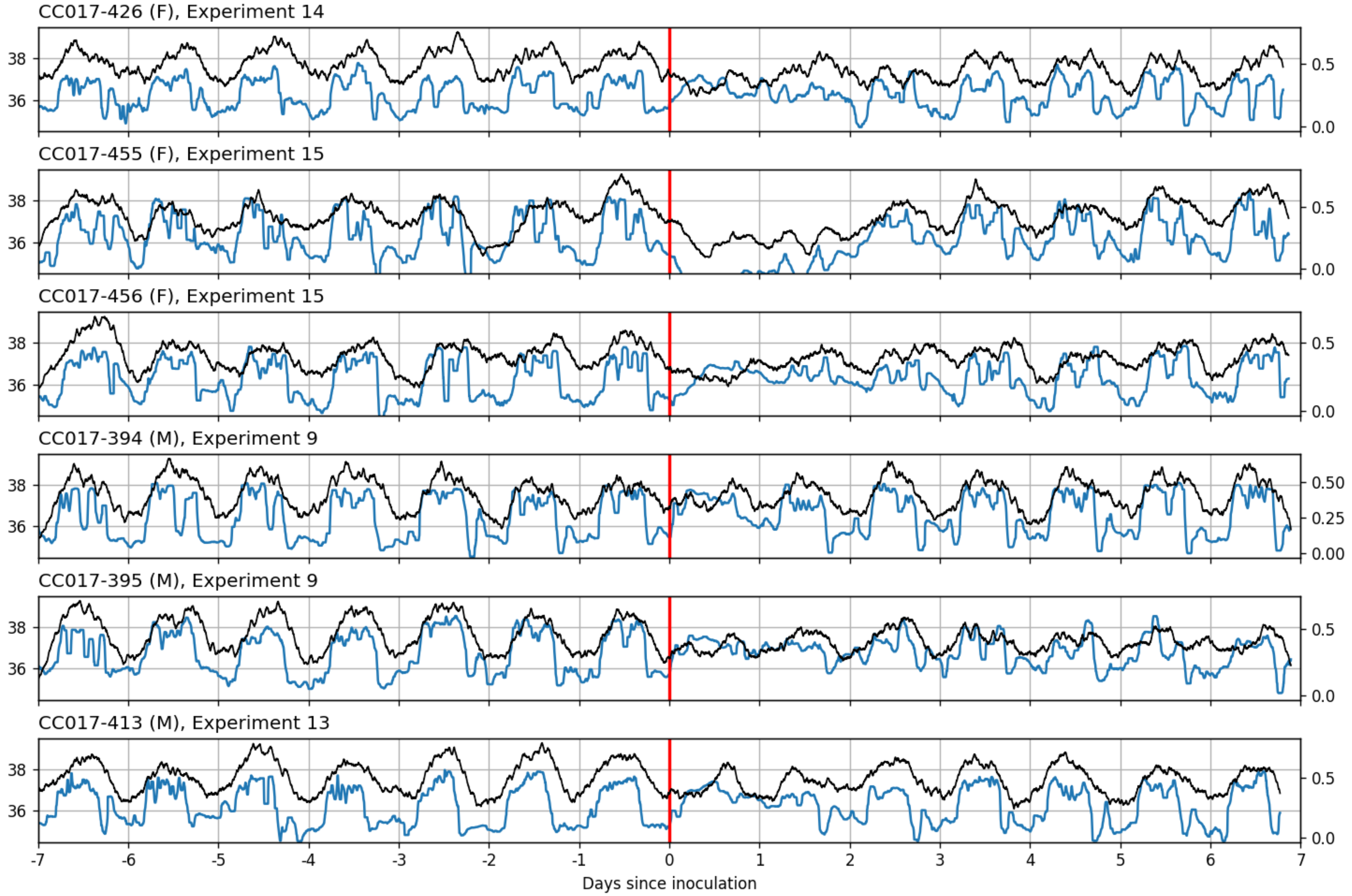

CC017-426 (F), Experiment 14
CC017-455 (F), Experiment 15
CC017-456 (F), Experiment 15
CC017-394 (M), Experiment 9
CC017-395 (M), Experiment 9
CC017-413 (M), Experiment 13
36
38
0.0
0.25
0.5
0.50
0.00
-7
-6
-5
-4
-3
-2
-1
0
1
2
3
4
5
6
7
Days since inoculation

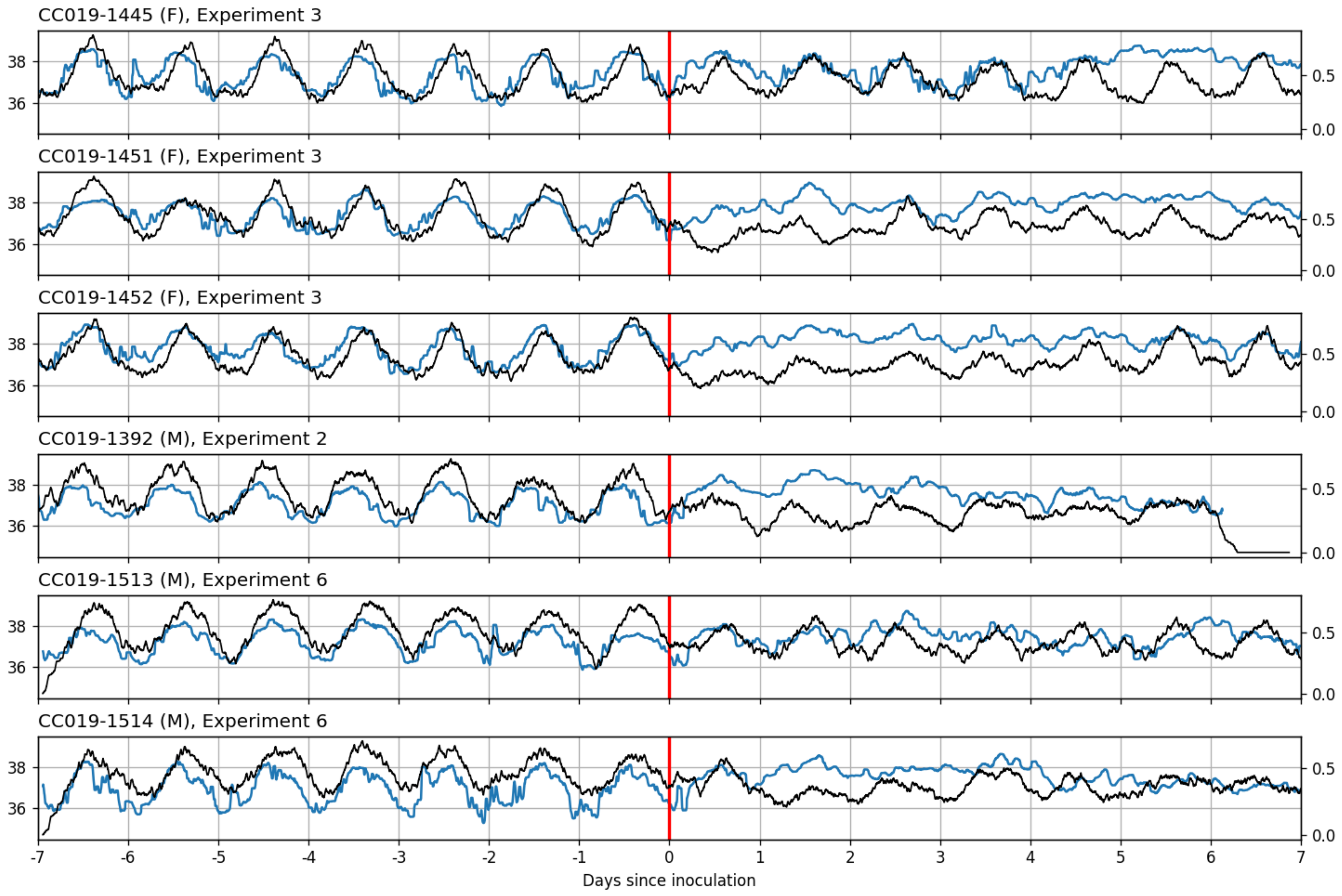
CC019-1445 (F), Experiment 3
CC019-1451 (F), Experiment 3
CC019-1452 (F), Experiment 3
CC019-1392 (M), Experiment 2
CC019-1513 (M), Experiment 6
CC019-1514 (M), Experiment 6
38
36
0.5
0.0
-7
-6
-5
-4
-3
-2
-1
0
1
2
3
4
5
6
7
Days since inoculation

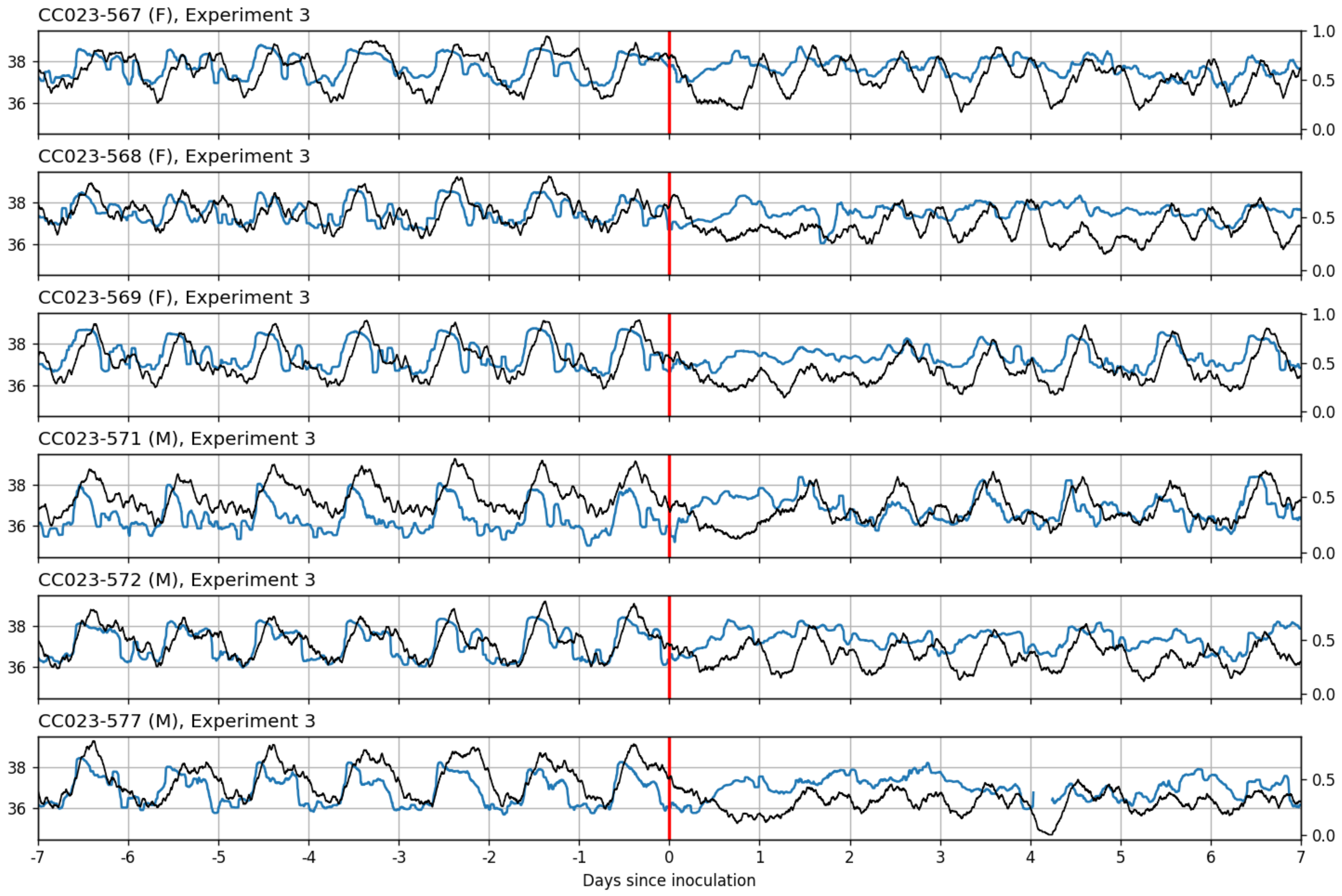

CC023-567 (F), Experiment 3
CC023-568 (F), Experiment 3
CC023-569 (F), Experiment 3
CC023-571 (M), Experiment 3
CC023-572 (M), Experiment 3
CC023-577 (M), Experiment 3
38
36
1.0
0.5
0.0
-7
-6
-5
-4
-3
-2
-1
0
1
2
3
4
5
6
7
Days since inoculation

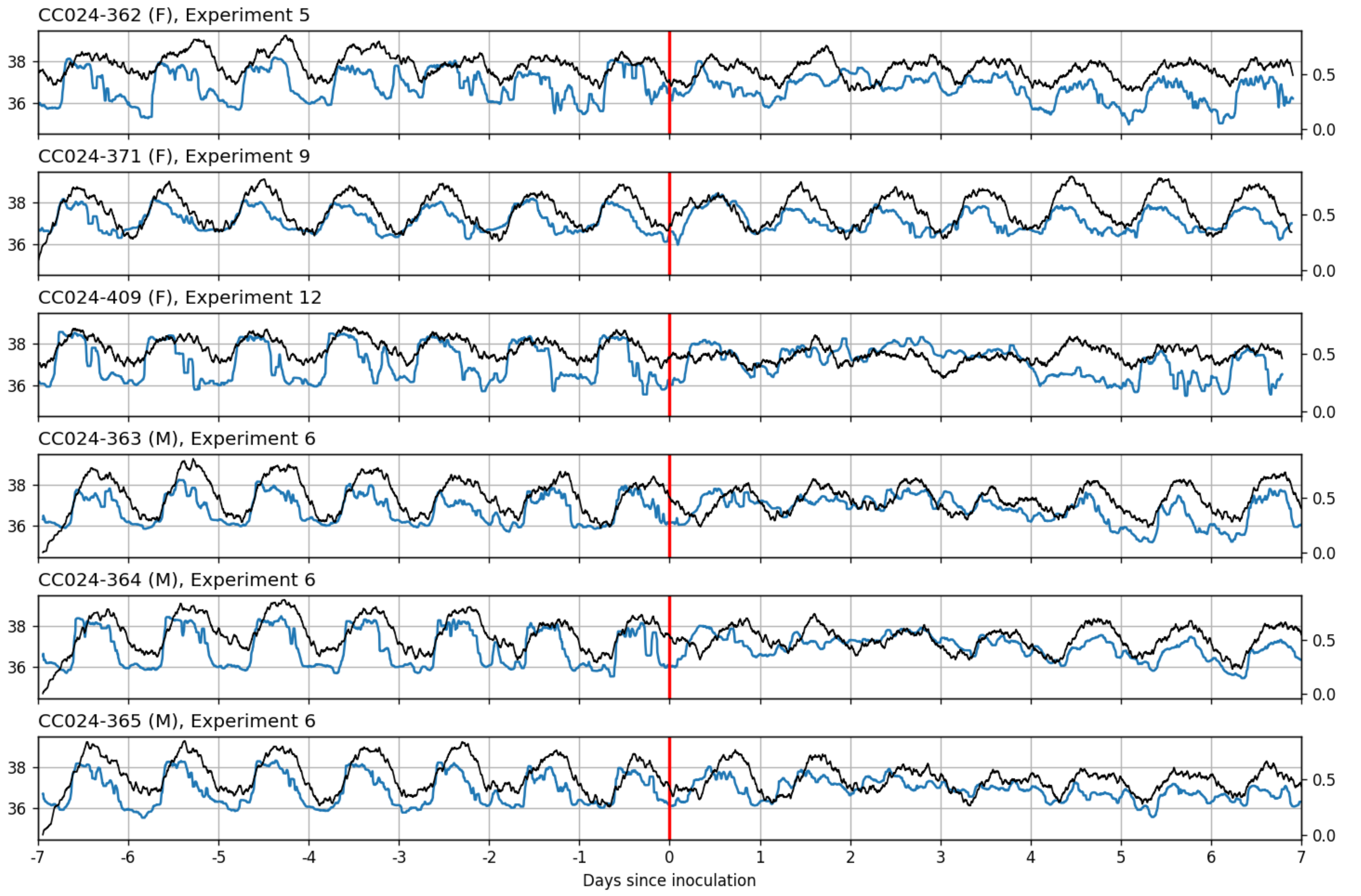
CC024-362 (F), Experiment 5
CC024-371 (F), Experiment 9
CC024-409 (F), Experiment 12
CC024-363 (M), Experiment 6
CC024-364 (M), Experiment 6
CC024-365 (M), Experiment 6
38
36
0.5
0.0
-7
-6
-5
-4
-3
-2
-1
0
1
2
3
4
5
6
7
Days since inoculation

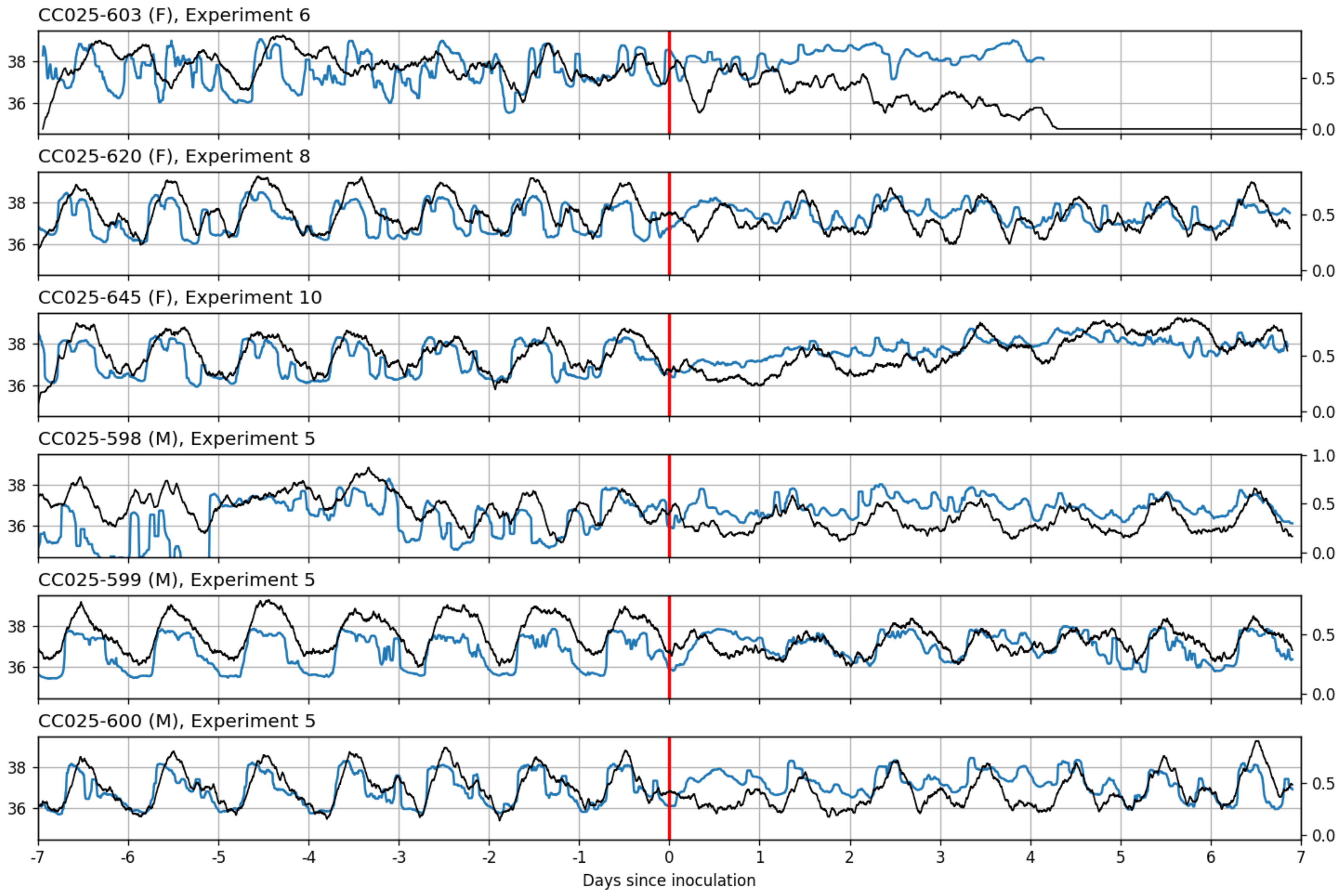

CC025-603 (F), Experiment 6
CC025-620 (F), Experiment 8
CC025-645 (F), Experiment 10
CC025-598 (M), Experiment 5
CC025-599 (M), Experiment 5
CC025-600 (M), Experiment 5
36
38
0.0
0.5
1.0
-7
-6
-5
-4
-3
-2
-1
0
1
2
3
4
5
6
7
Days since inoculation

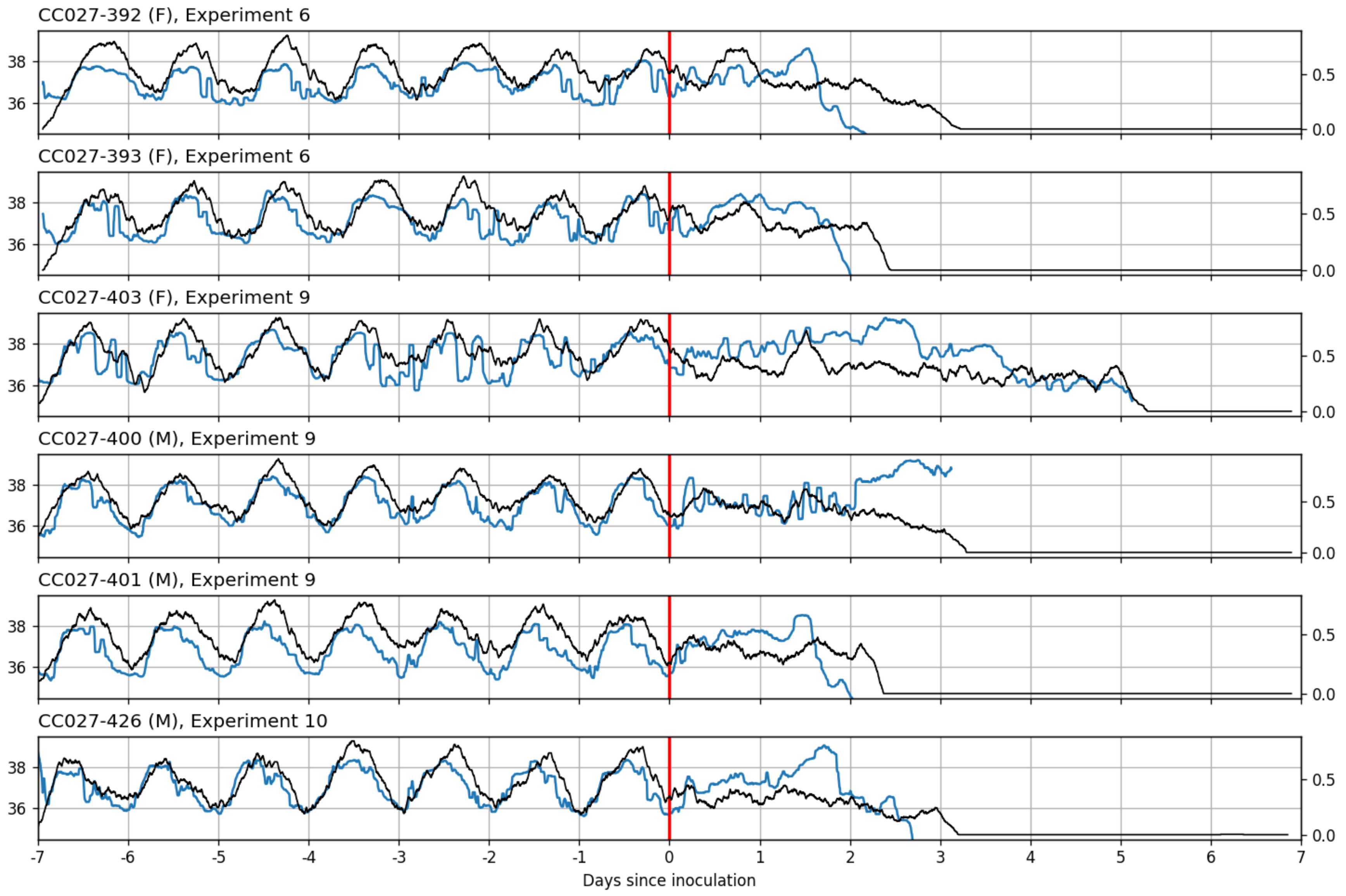

CC027-392 (F), Experiment 6
CC027-393 (F), Experiment 6
CC027-403 (F), Experiment 9
CC027-400 (M), Experiment 9
CC027-401 (M), Experiment 9
CC027-426 (M), Experiment 10
Days since inoculation

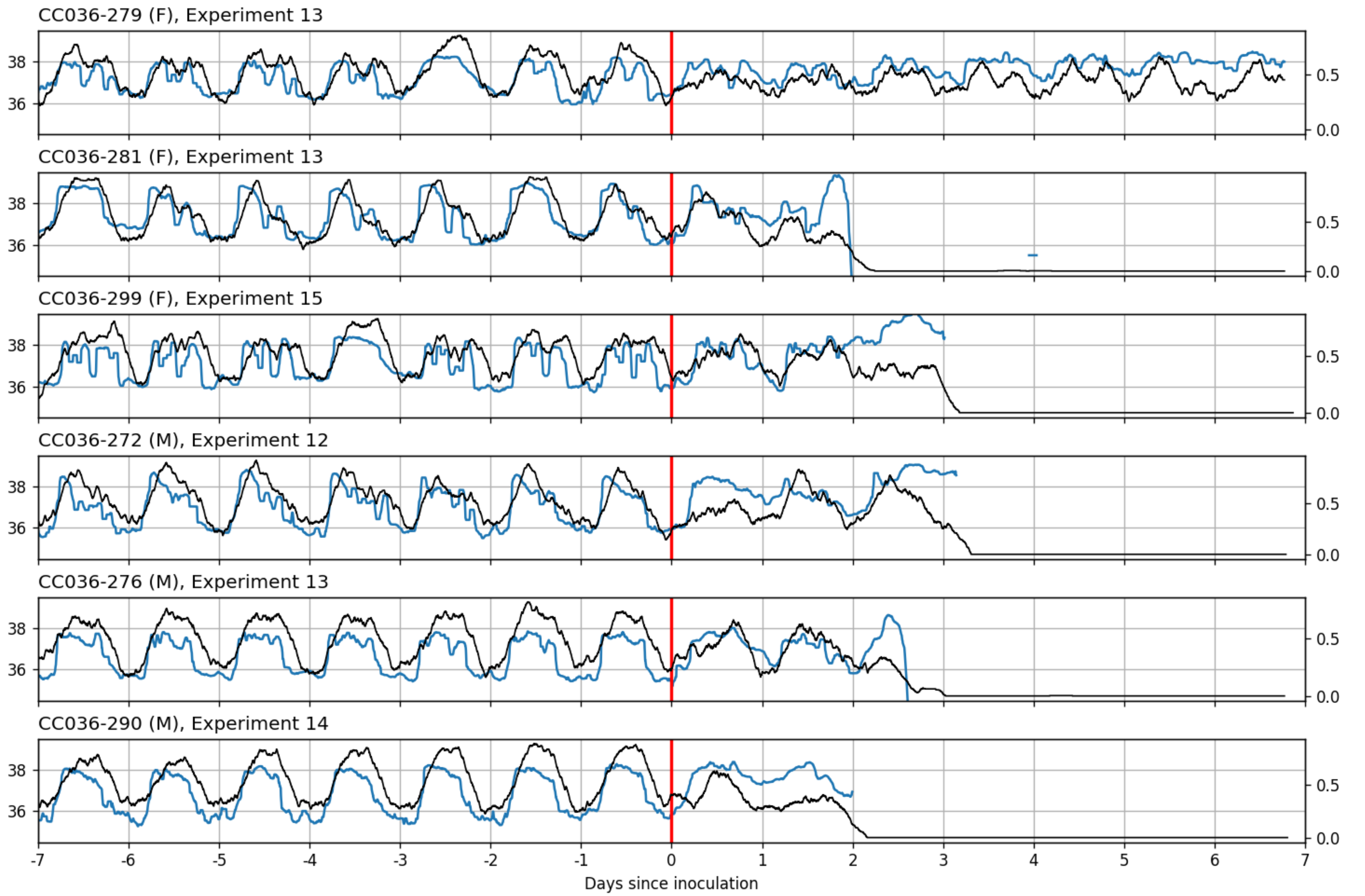

CC036-279 (F), Experiment 13
CC036-281 (F), Experiment 13
CC036-299 (F), Experiment 15
CC036-272 (M), Experiment 12
CC036-276 (M), Experiment 13
CC036-290 (M), Experiment 14
38
36
0.0
0.5
-7
-6
-5
-4
-3
-2
-1
0
1
2
3
4
5
6
7
Days since inoculation

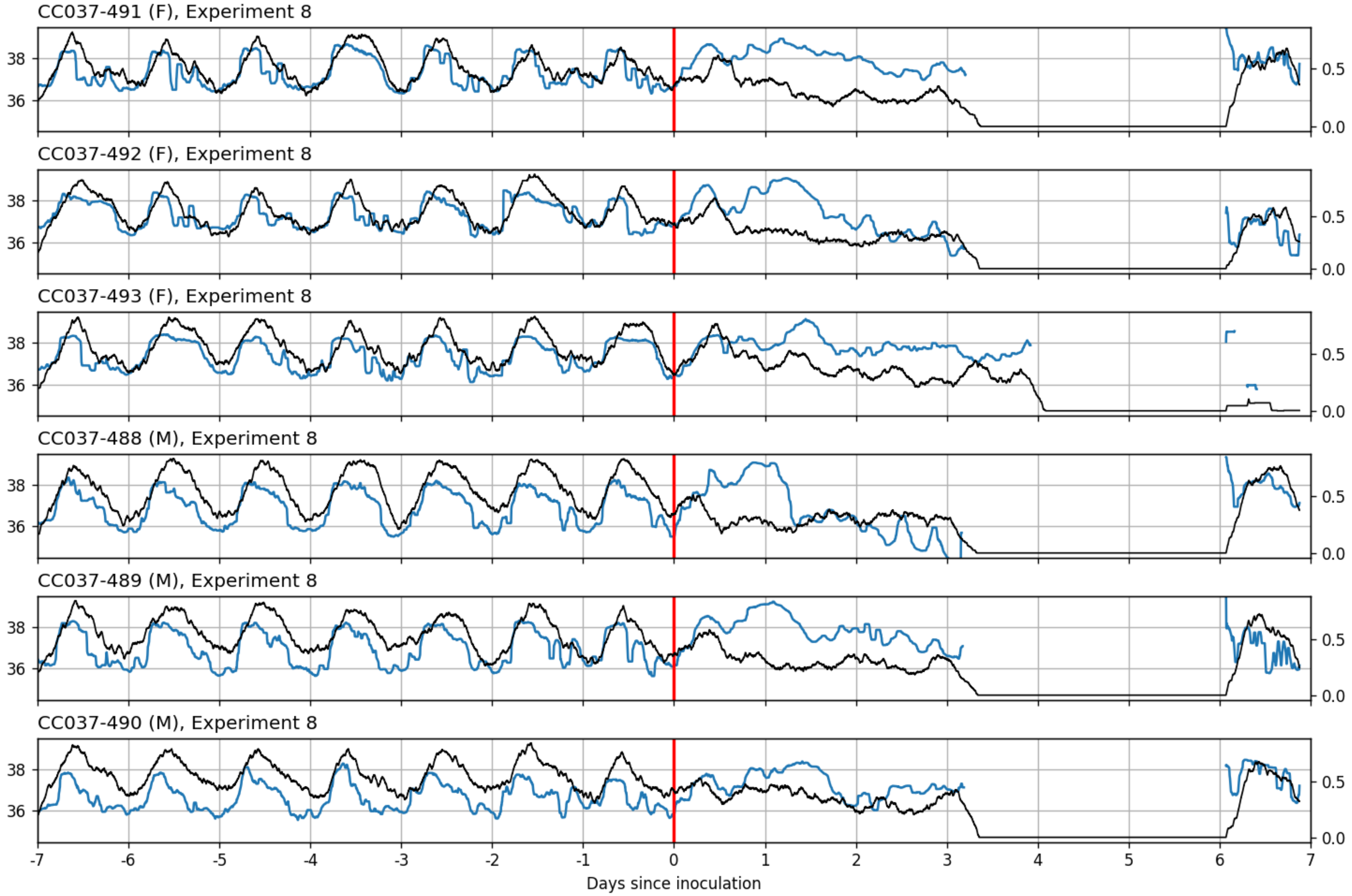
CC037-491 (F), Experiment 8
CC037-492 (F), Experiment 8
CC037-493 (F), Experiment 8
CC037-488 (M), Experiment 8
CC037-489 (M), Experiment 8
CC037-490 (M), Experiment 8
38
36
0.5
0.0
-7
-6
-5
-4
-3
-2
-1
0
1
2
3
4
5
6
7
Days since inoculation

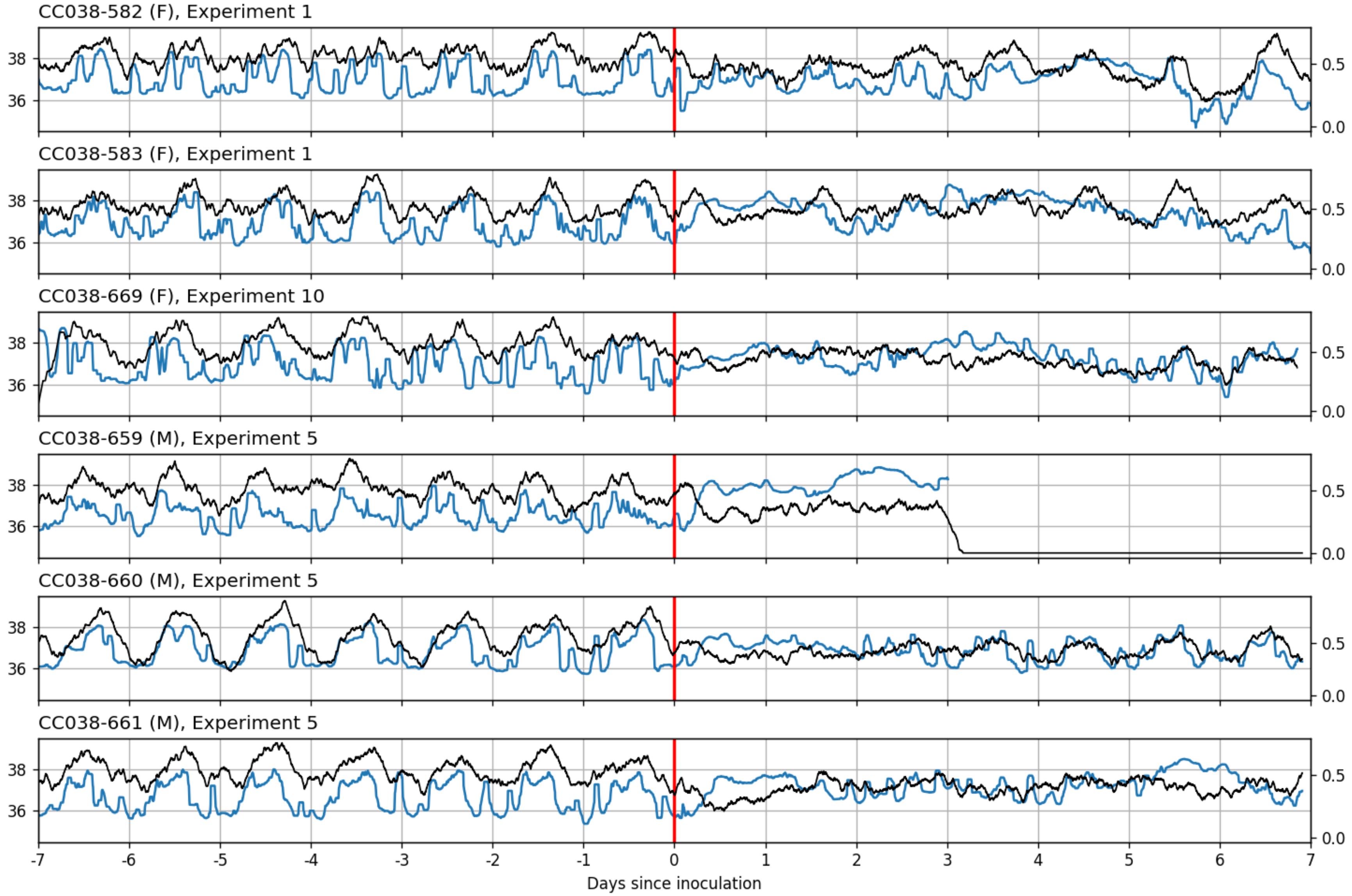

CC038-582 (F), Experiment 1
CC038-583 (F), Experiment 1
CC038-669 (F), Experiment 10
CC038-659 (M), Experiment 5
CC038-660 (M), Experiment 5
CC038-661 (M), Experiment 5
38
36
0.5
0.0
-7
-6
-5
-4
-3
-2
-1
0
1
2
3
4
5
6
7
Days since inoculation

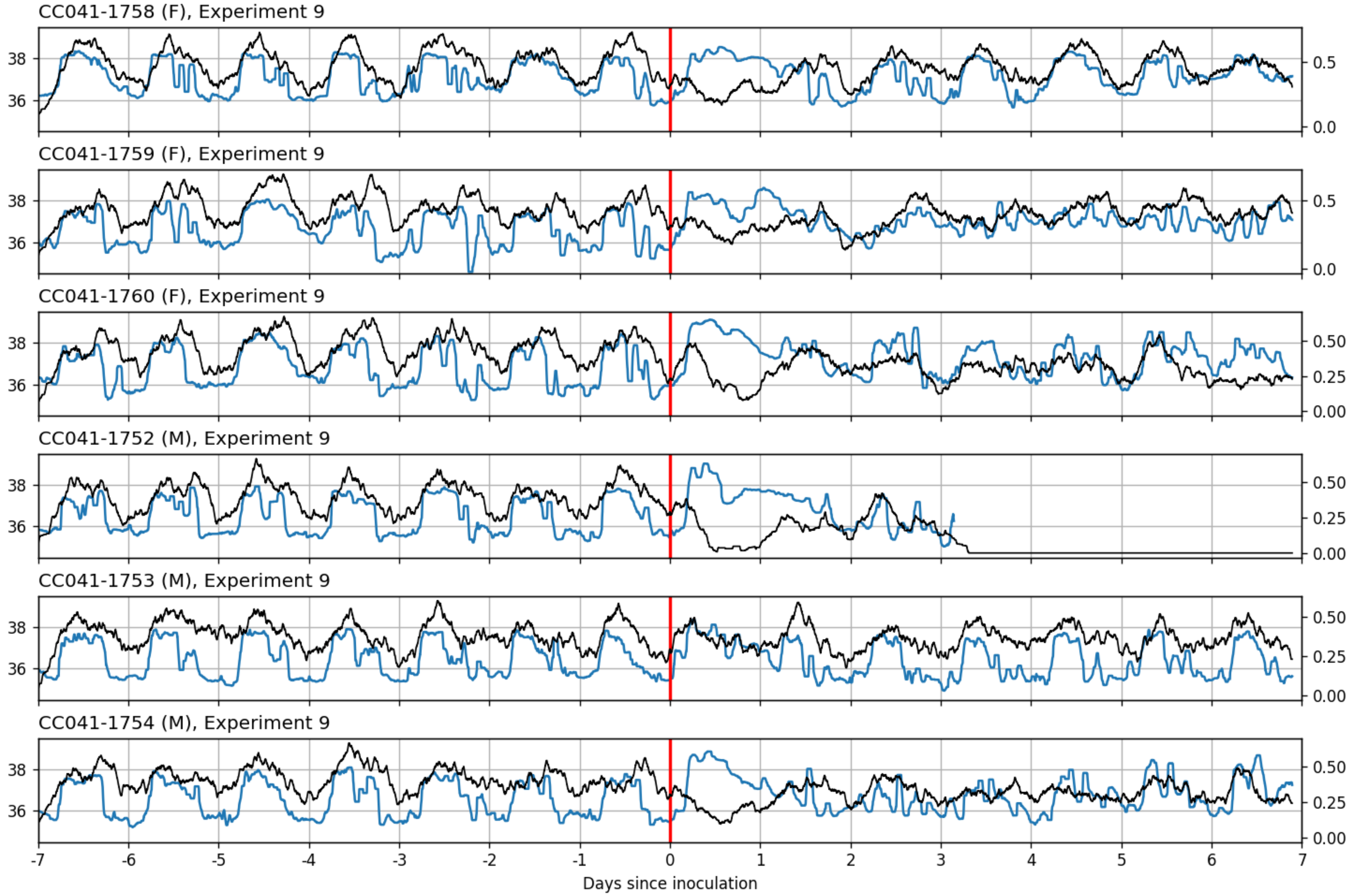
CC041-1758 (F), Experiment 9
CC041-1759 (F), Experiment 9
CC041-1760 (F), Experiment 9
CC041-1752 (M), Experiment 9
CC041-1753 (M), Experiment 9
CC041-1754 (M), Experiment 9
38
36
0.5
0.0
0.50
0.25
0.00
-7
-6
-5
-4
-3
-2
-1
0
1
2
3
4
5
6
7
Days since inoculation

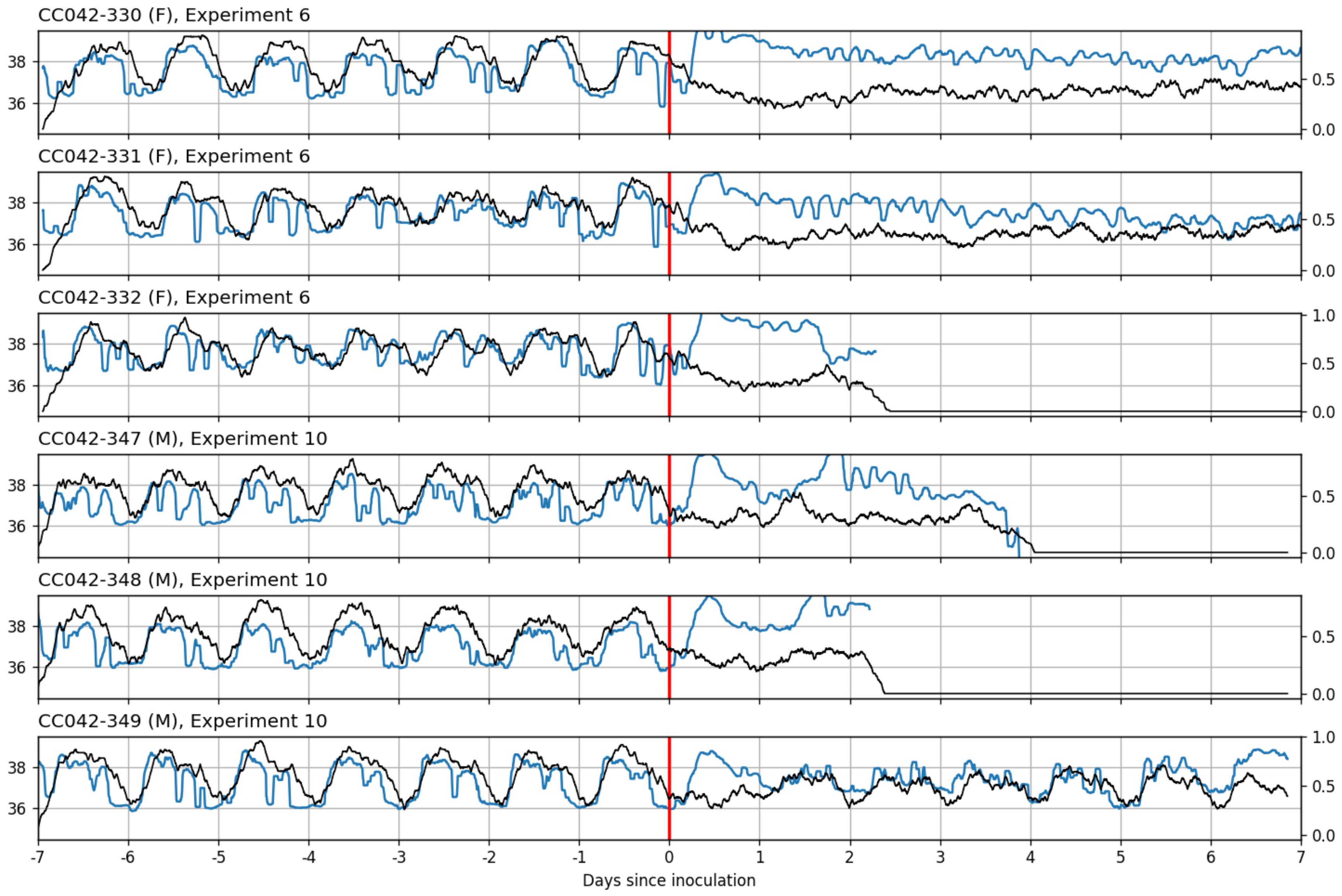
CC042-330 (F), Experiment 6
CC042-331 (F), Experiment 6
CC042-332 (F), Experiment 6
CC042-347 (M), Experiment 10
CC042-348 (M), Experiment 10
CC042-349 (M), Experiment 10
36
38
0.0
0.5
1.0
-7
-6
-5
-4
-3
-2
-1
0
1
2
3
4
5
6
7
Days since inoculation

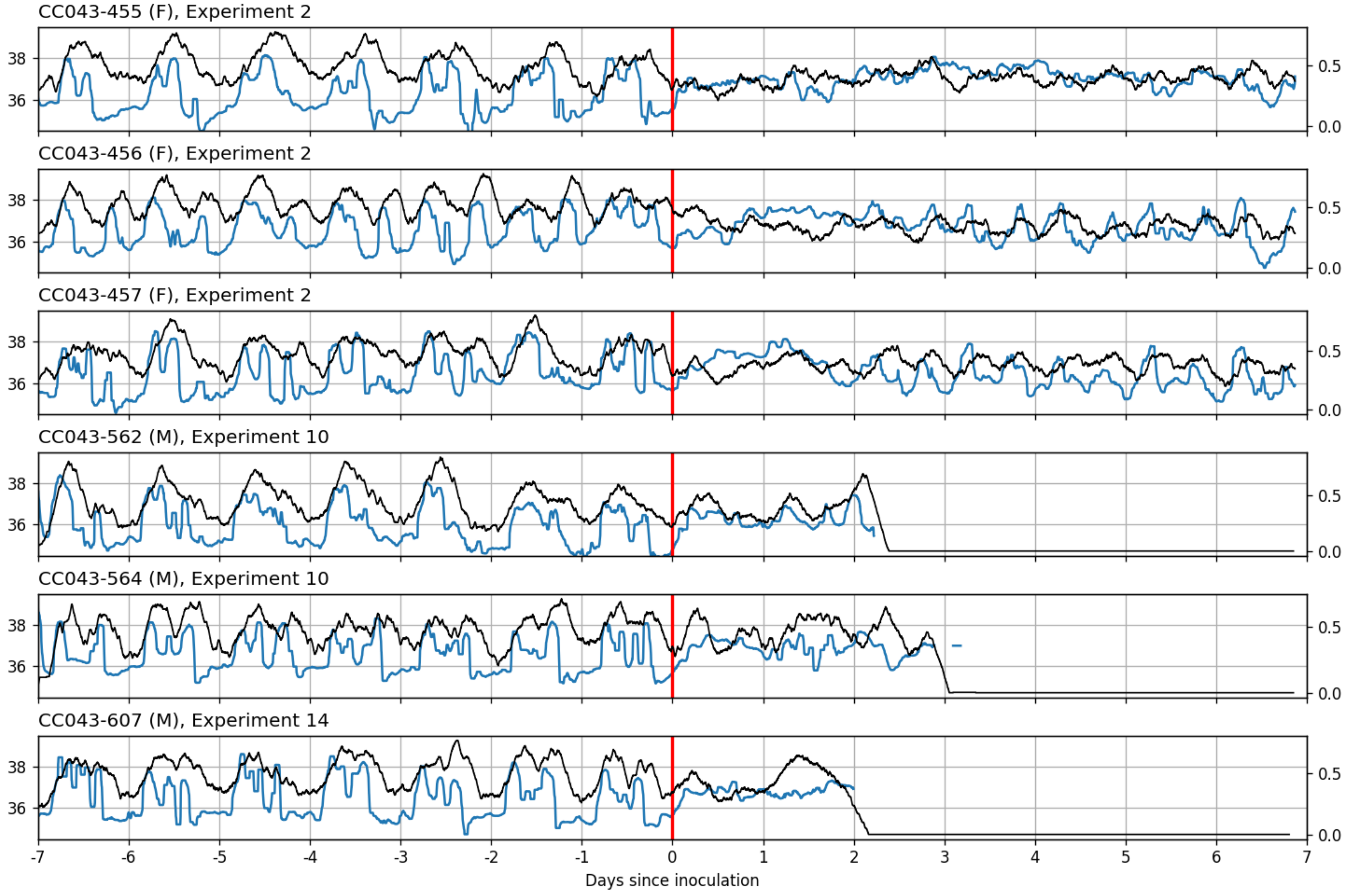
CC043-455 (F), Experiment 2
CC043-456 (F), Experiment 2
CC043-457 (F), Experiment 2
CC043-562 (M), Experiment 10
CC043-564 (M), Experiment 10
CC043-607 (M), Experiment 14
38
36
0.5
0.0
-7
-6
-5
-4
-3
-2
-1
0
1
2
3
4
5
6
7
Days since inoculation

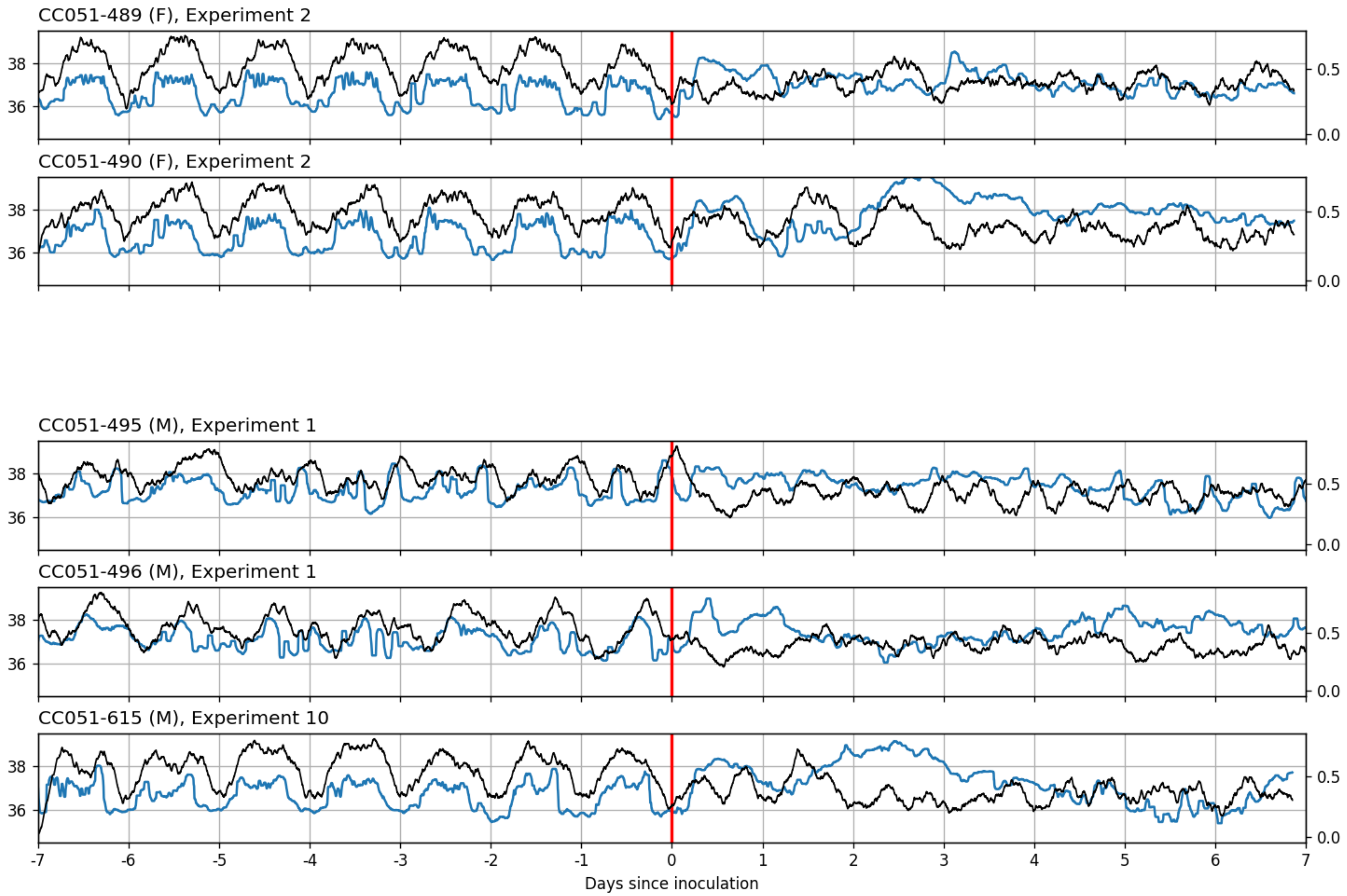
CC051-489 (F), Experiment 2
CC051-490 (F), Experiment 2
CC051-495 (M), Experiment 1
CC051-496 (M), Experiment 1
CC051-615 (M), Experiment 10
38
36
0.5
0.0
-7
-6
-5
-4
-3
-2
-1
0
1
2
3
4
5
6
7
Days since inoculation

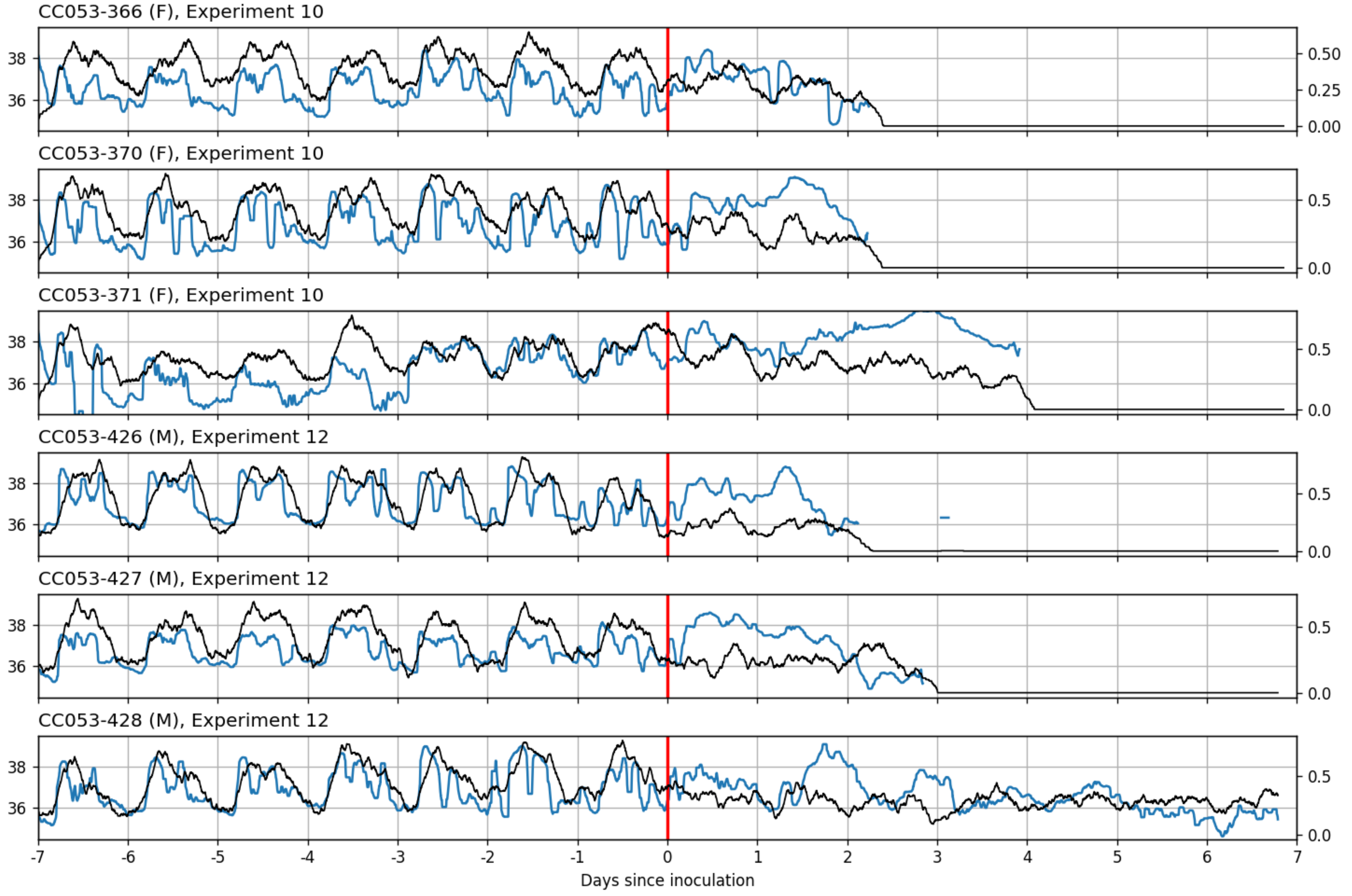

CC053-366 (F), Experiment 10
CC053-370 (F), Experiment 10
CC053-371 (F), Experiment 10
CC053-426 (M), Experiment 12
CC053-427 (M), Experiment 12
CC053-428 (M), Experiment 12
36
38
0.00
0.25
0.50
0.0
0.5
-7
-6
-5
-4
-3
-2
-1
0
1
2
3
4
5
6
7
Days since inoculation

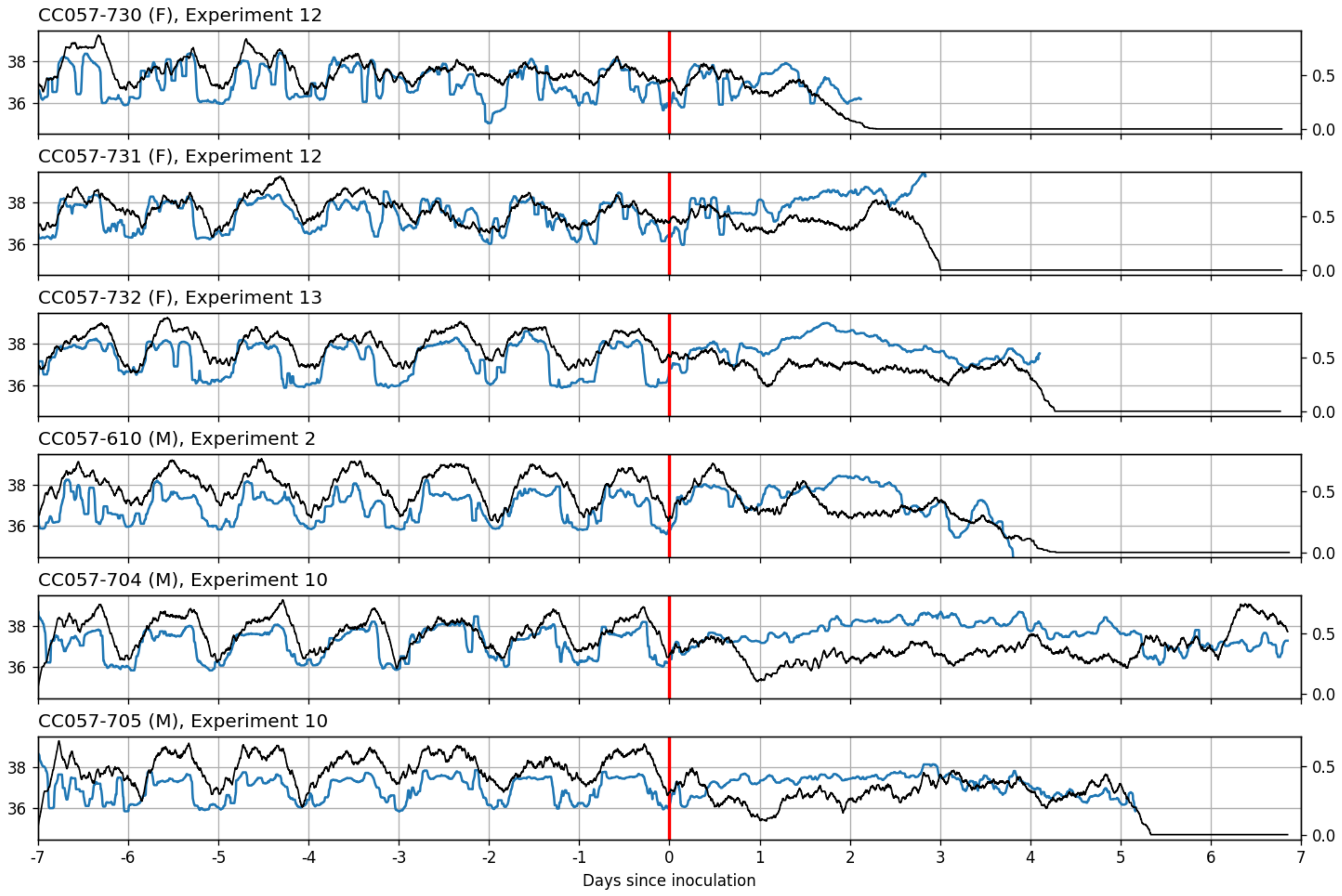

CC057-730 (F), Experiment 12
CC057-731 (F), Experiment 12
CC057-732 (F), Experiment 13
CC057-610 (M), Experiment 2
CC057-704 (M), Experiment 10
CC057-705 (M), Experiment 10
36
38
0.0
0.5
-7
-6
-5
-4
-3
-2
-1
0
1
2
3
4
5
6
7
Days since inoculation

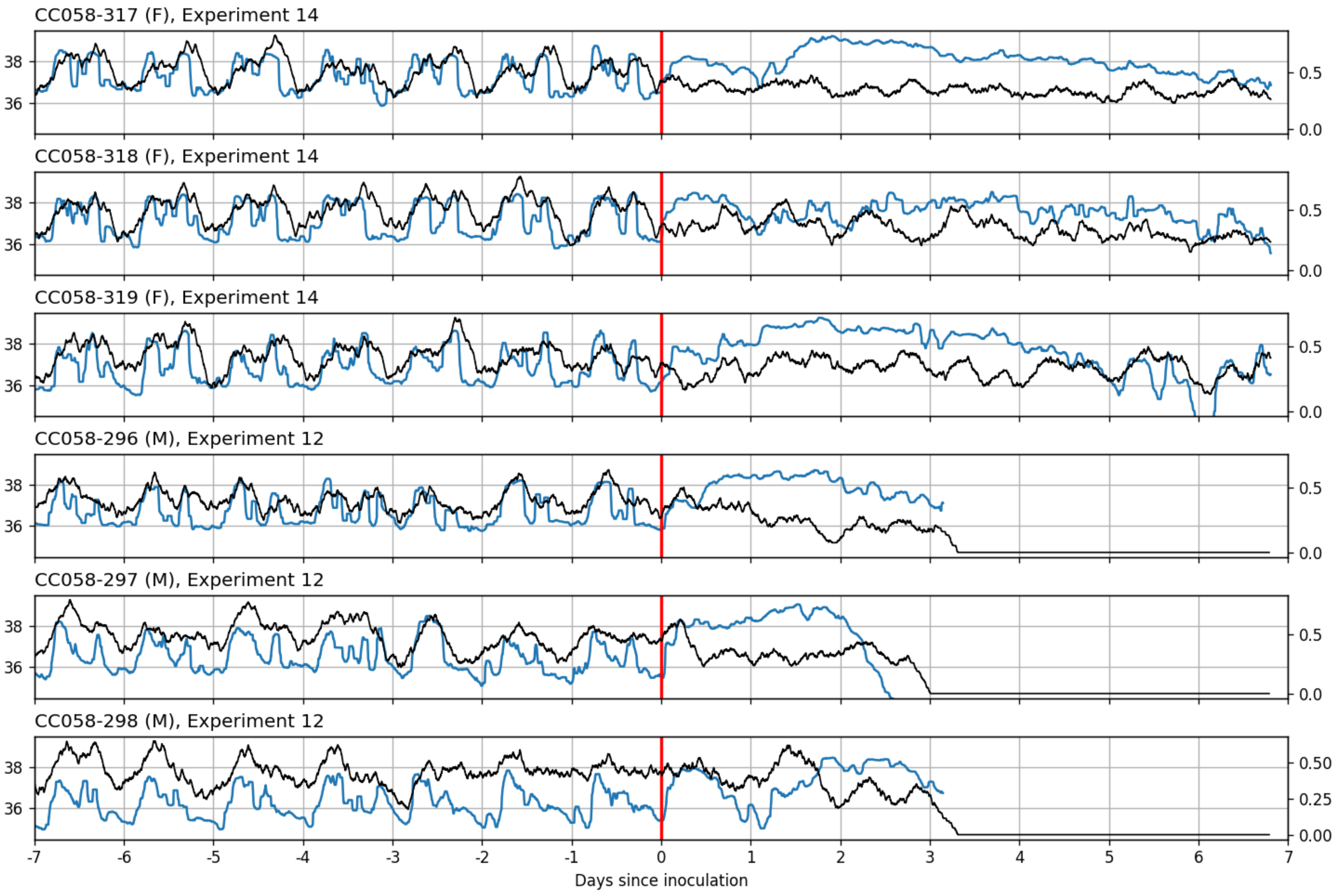
CC058-317 (F), Experiment 14
CC058-318 (F), Experiment 14
CC058-319 (F), Experiment 14
CC058-296 (M), Experiment 12
CC058-297 (M), Experiment 12
CC058-298 (M), Experiment 12
36
38
0.0
0.5
0.00
0.25
0.50
-7
-6
-5
-4
-3
-2
-1
0
1
2
3
4
5
6
7
Days since inoculation

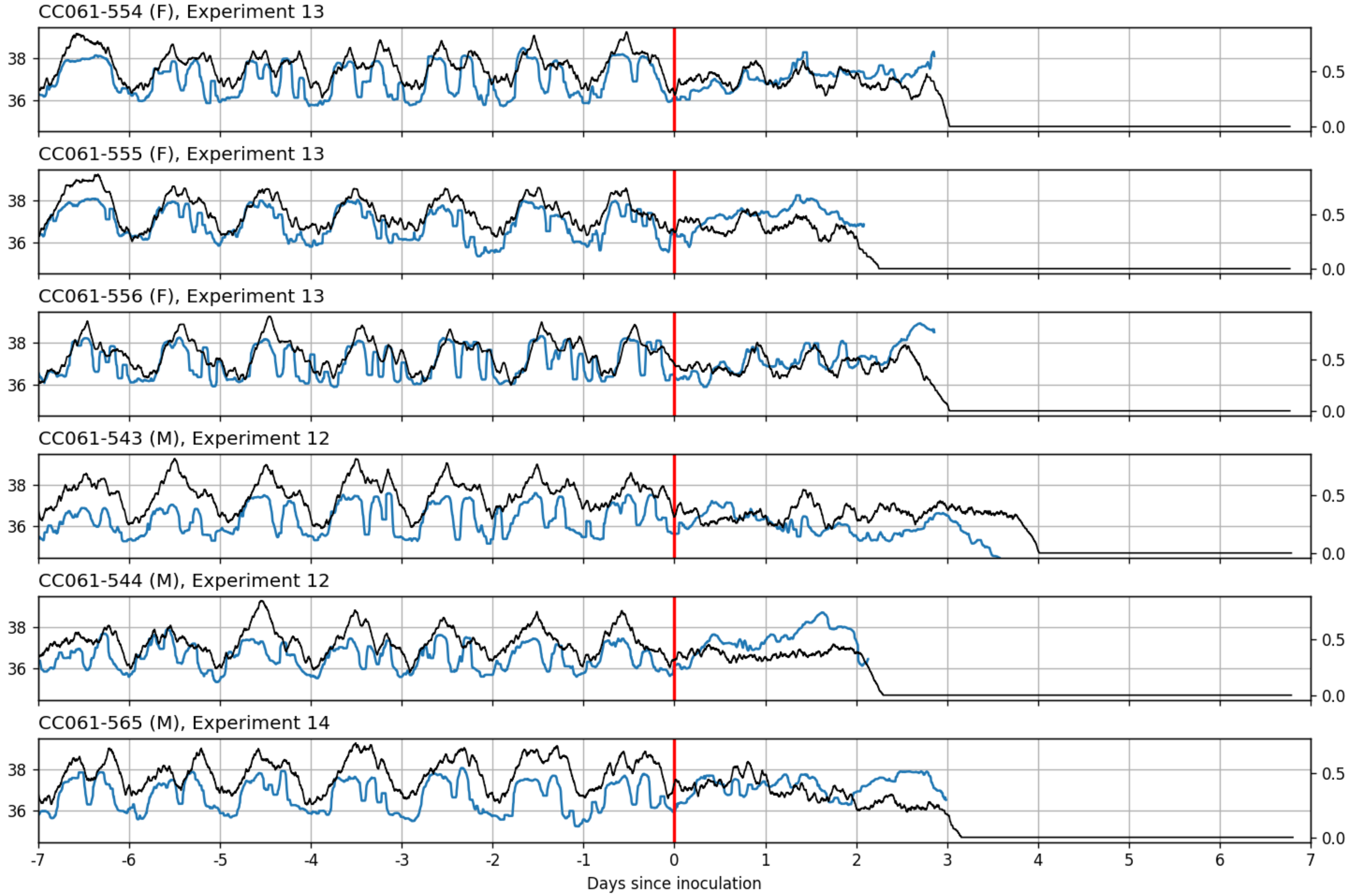
CC061-554 (F), Experiment 13
CC061-555 (F), Experiment 13
CC061-556 (F), Experiment 13
CC061-543 (M), Experiment 12
CC061-544 (M), Experiment 12
CC061-565 (M), Experiment 14
38
36
0.5
0.0
-7
-6
-5
-4
-3
-2
-1
0
1
2
3
4
5
6
7
Days since inoculation
